# Characterization of the extracellular vesicles, ultrastructural morphology, and intercellular interactions of multiple clinical isolates of the brain-eating amoeba, *Naegleria fowleri*

**DOI:** 10.3389/fmicb.2023.1264348

**Published:** 2023-09-27

**Authors:** A. Cassiopeia Russell, Peter Bush, Gabriela Grigorean, Dennis E. Kyle

**Affiliations:** ^1^Center for Tropical and Emerging Global Diseases, University of Georgia, Athens, GA, United States; ^2^Department of Infectious Diseases, University of Georgia, Athens, GA, United States; ^3^School of Dental Medicine, University at Buffalo, Buffalo, NY, United States; ^4^Proteomics Core Facility, University of California, Davis, Davis, CA, United States; ^5^Department of Cellular Biology, University of Georgia, Athens, GA, United States

**Keywords:** *Naegleria fowleri*, extracellular vesicles, ultrastructure, intercellular interactions, parasite

## Abstract

**Introduction:**

As global temperatures rise to unprecedented historic levels, so too do the latitudes of habitable niches for the pathogenic free-living amoeba, *Naegleria fowleri*. This opportunistic parasite causes a rare, but >97% fatal, neurological infection called primary amoebic meningoencephalitis. Despite its lethality, this parasite remains one of the most neglected and understudied parasitic protozoans.

**Methods:**

To better understand amoeboid intercellular communication, we elucidate the structure, proteome, and potential secretion mechanisms of amoeba-derived extracellular vesicles (EVs), which are membrane-bound communication apparatuses that relay messages and can be used as biomarkers for diagnostics in various diseases.

**Results and Discussion:**

Herein we propose that *N. fowleri* secretes EVs in clusters from the plasma membrane, from multivesicular bodies, and via beading of thin filaments extruding from the membrane. Uptake assays demonstrate that EVs are taken up by other amoebae and mammalian cells, and we observed a real-time increase in metabolic activity for mammalian cells exposed to EVs from amoebae. Proteomic analysis revealed >2,000 proteins within the *N. fowleri*-secreted EVs, providing targets for the development of diagnostics or therapeutics. Our work expands the knowledge of intercellular interactions among these amoebae and subsequently deepens the understanding of the mechanistic basis of PAM.

## 1. Introduction

*Naegleria fowleri* is a thermophilic free-living amoeba found ubiquitously in soil, fresh, and brackish waters (Fowler and Carter, 1965; Grace et al., 2015; Siddiqui et al., 2016; Xue et al., 2018). This amphizoic pathogen is the etiologic agent for the fulminant disease known as primary amoebic meningoencephalitis (PAM), a neurological illness that commonly affects young healthy individuals (Visvesvara et al., 2007; Capewell et al., 2015). Infections in humans occur when contaminated warm water enters the nose—usually during recreational water sports or nasal irrigation/ablutions—and the invasive trophozoite stage of the parasite binds to and colonizes the nasal epithelium (Visvesvara et al., 2007; Pugh and Levy, 2016). *N. fowleri* then travels through the nasal mucosa and along the neuro-olfactory nerves through the cribriform plate to reach the olfactory bulbs in the frontal lobes where it feeds on neurons and damages brain membranes and meninges (Barnett et al., 1996; Capewell et al., 2015; Grace et al., 2015; Siddiqui et al., 2016). Although the pathogenicity of the amoeba contributes to some of the damage, the host’s profound immune response ultimately leads to death due to increased intracranial pressure and brain herniation resulting in pulmonary edema and cardiopulmonary arrest (Visvesvara et al., 2007). The incubation period of PAM ranges from 2 to 15 days with >97% of cases resulting in death approximately one week after the initial appearance of symptoms (Ma et al., 1990; Siddiqui et al., 2016). Hundreds of cases have been documented worldwide with the majority of cases being reported in countries with developed medical systems (Visvesvara et al., 2007) including the United States, Australia, and Europe, thus it can be assumed that there are many more cases globally that go undiagnosed, mistaken for bacterial or viral meningitis, and unreported in less developed tropical regions. Though a rare infection, it most likely kills thousands worldwide, but is overlooked due to lack of routinely performed post-mortem examination of inexplicable neurological deaths. In the last 20 years, a rise in the number of reported PAM cases can be attributed to increased awareness and the onset of globalwarming (Kemble et al., 2012) leading to the expansion of suitable climates for *N. fowleri* (Booth et al., 2015; Cope and Ali, 2016).

Various proteins have been identified that aid *N. fowleri* in the attachment to host cells, in invasion of the host central nervous system (CNS), and in obtaining nutrients from target cells (Sohn et al., 2010; Jamerson et al., 2012; Lam et al., 2017), but little is known regarding the mechanism of secretion of vesicles containing these proteins and other cargoes. Extracellular vesicles (EVs), which are membrane-bound particles shed from cells, are known to carry cargo that consists of proteins, DNA, mRNAs, and microRNAs (van Niel et al., 2018; Malkin and Bratman, 2020). These particles, known as exosomes and microvesicles, merge with recipient cells and deliver their contents, thereby altering the function of the recipient cell (Mathieu et al., 2019). Numerous pathogens and eukaryotic parasites are known to secrete EVs to mediate host responses to infection and to communicate intercellularly (Marcilla et al., 2014; Quintana et al., 2017; Goncalves et al., 2018; Lin et al., 2019; Rai and Johnson, 2019). Additionally, recent reports show that *N. fowleri* secretes EVs (*Nf*-EVs) that are immunogenic, contain at least 200 proteins, and are taken up via phagocytosis by macrophages (Lertjuthaporn et al., 2022; Retana Moreira et al., 2022), but no research has been performed that explores the mechanism of EV release, or the uptake of *Nf-*EVs by amoebae or multiple mammalian cell lines. The uptake of secretions by multiple cell lines could be important because *N. fowleri* must traverse numerous types of cells before reaching the brain (Martinez et al., 1973). Moreover, little has been done to elucidate specific physical structures that amoebae may exhibit when in the process of invading, interacting with, or actively feeding upon mammalian cells versus typical morphology of axenically cultured amoebae. Information about specific morphological changes as well as the mechanism by which immunogenic *Nf*-EVs are secreted could be vital in illuminating novel structures and downstream processes to target by drugs and potential prophylactics.

Herein, we use scanning electron microscopy (SEM) to characterize the ultrastructural morphology of five clinical isolates of *N. fowleri* in both axenic culture and when feeding on various mammalian cell lines. Isolates of *N. fowleri* are known to have differences in drug susceptibilities and growth rate (Duma and Finley, 1976; Schuster et al., 2006; Russell and Kyle, 2022), thus we selected five of varying genotypes for further characterization in this study: *Nf69* (genotype IV/5), *V067* (III/3)*, HB4* (III/3)*, V631* (I/2), and *6088* (II/1). Additionally, we propose the secretion of clusters of *Nf-*EVs from the amoebae via multivesicular bodies, plasma membrane budding and a novel secretion technique in which clusters of uroid and/or adhesive filaments attach to the substrate and either break off from the cell in an intact form, or vesicularize in a beaded manner. Furthermore, we show the uptake of *Nf-*EVs by various mammalian cell lines and the real-time response of three of these cell lines when taking up *Nf-*EVs over time. Lastly, we provide a proteomic profile of >2,000 proteins found within *Nf*-secreted EVs which could be used as a resource for the development of novel therapeutics and protein-based diagnostics.

## 2. Materials and methods

### 2.1. *Naegleria fowleri* cultivation

All clinical isolates were obtained as previously described in Russell and Kyle (2022). Trophozoites were cultured axenically as previously described with some changes according to desired uses. Shortly, trophozoites were grown from stocks axenically at 34°C and 5% CO_2_ in non-vented 75-cm^2^ tissue culture flasks (Olympus, El Cajon, CA, United States; cat#:25-208) with Nelson’s complete medium (NCM) supplemented with 10% fetal bovine serum (FBS; Corning, Oneonta, NY, United States; cat#:35-016-CV) and 1,000 U/mL penicillin and 1,000 mg/mL streptomycin (penstrep; Gibco, Gaithersburg, MD, United States; cat#:15140-122) until 80–90% confluent.

### 2.2. Mammalian cell cultivation

All mammalian cells were grown in vented 75-cm^2^ tissue culture flasks (ThermoFisher, Waltham, MA, United States; cat#:156367) at 37°C and 5% CO_2_. A549 cells (Human lung carcinoma cells; ATCC CCL-185) were purchased from American Type Culture Collection (ATCC) and grown in F12K media (Corning, Oneonta, NY, United States; cat#:10-025-CV) supplemented with 10% FBS and 1% penstrep. B103 cells (rat neuroblastoma cells) were purchased from AddexBio (cat#:C0005003) and were grown in DMEM (Corning, Oneonta, NY, United States; cat#:10-013-CM) supplemented with 10% FBS and 1% penstrep. HFF cells (Human foreskin fibroblasts; HFF-1 ATCC SCRC-1041) were purchased from ATCC and were cultured in DMEM supplemented with 15% FBS and 1% penstrep. Vero cells (green monkey kidney cells; E6; ATCC CRL-1586) were purchased from ATCC and were cultured in DMEM supplemented with 10% FBS and 1% penstrep. For passaging, all cell types were first washed with pre-warmed PBS to remove residual serum and then incubated with pre-warmed 0.25% Trypsin–EDTA (Gibco, Gaithersburg, MD, United States; cat#:25200-056) for 5 min at 37°C. Following incubation, flasks were lightly tapped until all cells were detached and respective pre-warmed media supplemented with FBS was added to inactivate the trypsin prior to centrifugation at 37°C for 5 min at 3,900 rpm and resuspension in respective media.

### 2.3. Preparation of samples for SEM

For imaging axenic cultures, 5 × 10^5^ amoebae were allowed to attach to 13 mm Thermanox coverslips (Electron Microscopy Sciences, Hatfield, PA, United States; cat#:50-949-480) in 12-well plates prior to fixation. For feeding assays, 2.5–6 × 10^5^ mammalian cells were seeded into plates containing coverslips and allowed to attach/grow for 18–24 h before adding 3–6 × 10^5^ amoebae that were allowed to feed for various timepoints prior to fixation. Media was initially gently removed from samples and replaced with 2.5% glutaraldehyde (Electron Microscopy Sciences, Hatfield, PA, United States; cat#:16220) diluted in respective base media (no FBS or antibiotics added) and incubated for 1 h at RT. This was replaced with 2.5% glutaraldehyde diluted in 0.1 μm filtered 1X PBS (Gibco, Gaithersburg, MD, United States; cat#:10010-023) and incubated for 10 min at RT prior to being washed with PBS two times, each with a 10 min incubation period. Samples were then incubated for 5 min each with serially increasing concentrations (30, 50, 70, 90%) of molecular biology grade absolute ethanol (EtOH; Fisher BioReagents, Pittsburgh, PA; cat#:BP2818-500) diluted in 0.1 μm filtered MilliQ H2O. This was followed by two incubation periods of 5 min each in 100% EtOH. A final incubation step of 5 min in hexamethyldisilazane (Electron Microscopy Sciences, Hatfield, PA, United States; cat#:16700) was performed, liquid was removed, and samples were allowed to air dry in fume hood for 30 min – 1 h before being mounted on carbon-conductive tape (Ted Pella, Redding, CA, United States; cat#:16084-8) on microscope slides. Samples were transported to SEM facility, carbon-coated and imaged with a Hitachi SU70 scanning electron microscope at 2 kV. For preparation of EVs for imaging, EVs were diluted in 1 mL of 0.1 μm-filtered 1X PBS and this dilution was passed over a 13 mm 0.2 μm Whatman Nucleopore Track Etch Membrane (Cytiva, Marlborough, MA, United States; cat#:10417001) mounted in a 13 mm Swinnex Filter Holder (MilliporeSigma, Burlington, MA, United States; cat#:SX0001300) using a 3 mL syringe. Following this step, a new syringe containing 2.5% glutaraldehyde in 0.1 μm filtered PBS was attached and some of the contents were passed over the filter before capping and allowing to rest for 2 h at 4°C. Subsequent steps through imaging were performed in parallel with previously described samples in plates starting at 2.5% glutaraldehyde in PBS 10 min incubation step. Measurements were either performed upon pre-calibrated micrographs using the Quartz PCI SEM software (v8) or by manually calibrating to scale bars with ImageJ (v1.53k). Figures were generated using Adobe Photoshop (v23.5.2).

### 2.4. EV extraction

#### 2.4.1. Conditioned media preparation

To generate conditioned media for EV extractions, amoebae were extracted from flasks containing NCM supplemented with normal FBS by placing flasks on ice for ~15 min to detach adherent cells which were collected via centrifugation at 4°C for 5 min at 4,000 rpm. The resulting supernatant was discarded, and the amoebae pellets were washed two times with 0.1 μm-filtered 1X PBS to remove any residual FBS EVs or proteins. These washed cells were then placed into a non-vented 225-cm^2^ tissue culture flask (Corning, Oneonta, NY, United States; cat#:431081) containing 2× 0.1 μm filtered ~50 mL of Nelson’s Complete Media supplemented with 2× 0.2 μm filtered 10% EV-depleted FBS (Gibco, Gaithersburg, MD, United States; cat#:A2720801) and 5% penstrep and allowed to adapt to the differing conditions (with media changes as needed to remove debris/dead cells) and grow until ~80–90% confluent. This flask was then passaged to 5–10,225-cm^2^ flasks each containing ~200 mL of NCM supplemented with 10% EV-depleted FBS and 5% penstrep and these flasks were allowed to grow for 5–15 days with daily gentle swirling/agitation to induce growth until a peak yield was reached (based on morphology/visual inspection of health of amoebae) and flasks were > 90% confluent. To harvest cells and conditioned media, flasks were placed on ice for 30 min to thoroughly cool the contents, and the cell suspensions were spun at 3,900 rpm for 15 min at 4°C in 500 mL bottles (VWR, Radnor, PA, United States; cat#:525-1598). The resulting cell pellet was separated, and all pellets were combined for a final count (in duplicate) via hemocytometer for each prep. The supernatant was removed and spun for an additional 15 min at 10,000 g at 4°C to remove remaining cellular debris. The final supernatant was then sterile filtered through a 0.45 μm filter (ThermoFisher, Waltham, MA, United States; cat#:167-0045) to remove any remaining large particles or aggregates and create amoeba-conditioned media that was stored at 4°C for no more than 48 h prior to ultracentrifugation.

#### 2.4.2. Ultracentrifugation protocol

Using 70 mL polycarbonate centrifuge tubes (Beckman Coulter, Brea, CA, United States; cat#:355655), conditioned media was spun using a Ti45 fixed-angle rotor in an Optima XE-90 ultracentrifuge at ~118,000 g/39,000 rpm for 1 h 34 min with max acceleration and deceleration at 4°C. Additional spins were performed as needed by pouring off supernatant and adding remaining conditioned media until the entire volume was processed into a pellet. The resulting pellet was washed for 30 min at 4°C in 2–3 mL of ice-cold 0.1 μm sterile-filtered 1X PBS on a shaker, followed by another spin and one final wash/spin to remove secreted proteins and other components. After the final spin, the supernatant was discarded, and the EV pellet was resuspended in 3 mL of 1X PBS by incubating at 4°C on a shaker for 45 min-1 h. The resulting EV suspension was then concentrated down to 500 μL using a Amicon Ultra-4 3 kDa centrifugal filter unit (MilliporeSigma, Burlington, MA, United States; cat#:UFC800396) that was previously primed with 3 mL of PBS that was centrifuged out to remove particulates from the filter.

#### 2.4.3. Size exclusion chromatography

Size Exclusion Chromatography was then performed on the 500 μL concentrated suspension to clean up sample and remove secreted proteins using a qEVoriginal 70 nm column (Izon Science, Christchurch, New Zealand) according to manufacturer protocols. In short, column was first equilibrated to room temperature and then washed with three column volumes (10 mL each) of sterile 0.1 μm filtered 1X PBS. The concentrated 0.5 mL suspension from the ultracentrifugation protocol was then added to the column followed by 2.5 mL of PBS. The next 3–4 fractions (each consisting of 0.5 mL) were then collected depending on whether a purer EV suspension was desired or a higher yield. These fractions were pooled and transferred to another Amicon Ultra-4 3 kDa centrifugal filter and concentrated to ~100–250 μL according to manufacturer’s protocols.

#### 2.4.4. EV protein concentration measurement

Protein contents of EVs were measured using the Bio-Rad Protein Assay Kit II (BioRad, Hercules, CA, United States; cat#:5000002) according to the manufacturer’s microtiter plate protocol. Briefly, 5 μL of the 100–250 μL EV suspension plus 5 μL of 0.1 μm filtered 1X PBS was added to Corning 96-well clear polystyrene microplates (MilliporeSigma, Burlington, MA, United States; cat#:CLS3370) in duplicate, and a serial dilution of the provided bovine serum albumin (BSA) was performed in triplicate with 10 μL of each standard being added to three wells each prior to adding 200 μL of the dye reagent solution. Absorbance was measured after a 5 min incubation at 595 nm using a SpectraMax I3X plate reader (Molecular Devices, Sunnyvale, CA, United States), and protein concentrations of samples were determined by creating a standard curve of the BSA.

#### 2.4.5. Nanoparticle tracking analysis

A NanoSight NS300 (Malvern Pananalytical, Malvern, United Kingdom) instrument equipped with a syringe pump and utilizing NTA software v3.2 was used to determine particle size and concentration according to the following protocol. While 3 mL of 0.1 μm filtered 1X PBS was running through the machine with an infusion rate of 1,000, a 1:100 (10 μL EVs in 990 μL PBS) dilution was made before loading sample into the syringe pump and allowing ~300 μL to run through the line until particles were visible under camera. In the case of a highly concentrated sample in which particles are not easily identifiable and differentiated from one another, a new dilution of 1:1000 (1 μL of EVs in 999 μL PBS) was created and tested. A camera level of 14 was used for 10 captures with durations of 60 s each with a syringe pump infusion rate of 100. Once captures were collected, a detection threshold of 4 to 5 was selected depending on sensitivity required to detect the majority of particles in sample frames prior to data processing.

#### 2.4.6. SDS-PAGE gel

Protein contents of EVs were visualized on SDS PAGE gels prior to sending samples for mass spectrometry to confirm concentration and expected complexity of EV proteome. To obtain protein lysates of serial dilutions of amoebae, cell pellets were subjected to three freeze–thaw cycles (−80°C to 37°C) prior to resuspending in PBS. EV and cell lysate samples were first mixed with 4x Laemmli Sample Buffer (BioRad, Hercules, CA, United States; cat#:1610747) that was pre-mixed 1:10 (v/v) with 2-mercaptoethanol according to manufacturer protocols. Samples were then vortexed for 3 s prior to incubating at 70°C for 10 min followed by another 3 s vortex. Mini-PROTEAN 4–15% TGX Stain-Free protein gels (BioRad, Hercules, CA, United States; cat#:4568085) were prepared by rinsing with diH2O prior to submerging in SDS-PAGE running buffer and manually rinsing each well of the gel. Samples, 1 μL of BenchMark Protein Ladder (Invitrogen, Waltham, MA, United States; cat#:10747012) or blanks (sample buffer with PBS) were loaded to each well and gel was run at 100 V first for 2–3 min followed by 200 V for 20–30 min prior to imaging with a Bio-Rad ChemiDoc Imaging System.

### 2.5. Fluorescence microscopy of nanotubes

Amoebae were grown on glass coverslips under normal culturing procedures in 6-well plates until ~70–80% confluent. For fluorescence microscopy, samples were first fixed with 4% PFA and 0.5% glutaraldehyde for 15 min followed by a wash with PBS and a subsequent incubation with 2 μg/mL Hoescht 33342 (Invitrogen, Waltham, MA, United States; cat#:H21492) for 30 min. This was followed by another wash and a subsequent incubation step with 4 μL of the 1,000X DMSO suspension of SPY620-actin stain (Cytoskeleton Inc., Denver, CO, United States; cat#:CY-SC505) in a 1 mL stain solution for 30–45 min. A final wash with PBS was performed prior to mounting and imaging with either the DeltaVision II (pd20621) microscope or the Carl Zeiss Elyra 7 microscope. For the DeltaVision II, we show the maximum intensity projection of z-stacks imaged with the 100X objective that were deconvolved using the SoftWorx software (settings: enhanced ratio(aggressive), 10 cycles, medium(200 nm) noise filtering). For the Zeiss Elyra 7, a z-stack of 106 slices (9.546 μm) was obtained with the 63X objective and the Lattice SIM^2^ reconstruction algorithm was used to reconstruct the images and generate a 3D rendering (Grating Period: 617.32 nm, Processing: 3D, Input SNR: Medium, Iterations: 16, Regularization Weight: 0.065, Processing and Output Sampling: 4, Filter: Median, Detrend: No, Sectioning: 100, Baseline: Yes).

### 2.6. R18 EV uptake assays

#### 2.6.1. R18 EV labeling and excess dye removal

Octadecyl rhodamine B chloride (R18; Biotium, Fremont, CA, United States; cat#:60033) was diluted from 10 mM to 1 mM in DMSO (5 μL in 45 μL DMSO). EV suspensions that were split into 20 μg aliquots and stored at −80°C until use were allowed to thaw on ice, and PBS was added to reach a volume of 1 mL. 1 μL of 1 mM R18 was added to each tube for a final concentration of 1 μM. In parallel, 1 μL of 1 mM R18 was added to 999 μL of PBS as a control which was treated the same as samples from this step forward. All sample tubes were covered in foil to protect from light and incubated at 4°C overnight. The next morning, two PD-10 desalting columns (Cytiva, Marlborough, MA, United States; cat#:17-0851-01) were equilibrated per sample with 25 mL of 0.1 μm filtered 1X PBS. R18:vesicle solutions were passed through an equilibrated column by first adding the 1 mL suspension followed by 1.5 mL 1X PBS before eluting with 3.5 mL 1X PBS directly into a pre-washed Amicon Ultra-4 3 kDa centrifugal filter. These samples were concentrated to 1 mL before passing through a second desalting column and being concentrated to a final volume of 500 μL (equivalent to 1 μg of protein in 25 μL). Dyed EVs and control samples were stored in dark at 4°C until use (within 24 h).

#### 2.6.2. Deltavision high resolution imaging

3 × 10^5^ B103 cells were seeded onto glass coverslips in 6-well plates and allowed to attach and grow under normal growth conditions for 24 h. Media was then removed, and cells were washed once with 1 mL of pre-warmed serum-free DMEM prior to adding 2 mL of serum-free DMEM and serum starving cells for at least 1 h prior to EV treatment. All media was removed and replaced with ~500 μL of prewarmed serum-free DMEM (or enough to just coat glass coverslip) and 25 μL (equivalent to 1 μg) of R18-stained EVs were added to glass coverslips for various timepoints. At end of timepoint, samples were carefully washed thrice with prewarmed PBS prior to a combined stain/fix step with 4% PFA and 10 μg/mL Hoescht 33342. Coverslips were mounted on glass slides and sealed with clear nail polish prior to being imaged at 100X with the DeltaVision I (pd125225) Olympus IX-71 inverted microscope. Images were obtained with consistent percent transmission and exposure settings regardless of timepoint. Images were acquired as z-stacks that were deconvolved using the SoftWorx software (settings: enhanced ratio(aggressive), 10 cycles, medium(200 nm) noise filtering). The scaling for image/wavelength attributes of each channel for the maximum intensity projections were edited to the same values for every exported photo (DAPI = 82/872/1; TRITC = 51/1044/1) to reflect any changes in fluorescence intensity consistently without introducing bias.

#### 2.6.3. High-content imaging of R18-stained EV uptake by mammalian cells

Cells were seeded at 10,000 cells per well for A549, 7,500 cells per well for HFF, and 5,000 cells per well for B103 and Vero into μClear black Cellstar 96-well microplates (Greiner Bio-One, Kremsmünster, Austria; cat#:655090) in 100 μL of their respective media per well. The next morning, media was removed and replaced with respective serum-free base medias to serum starve cells for 1–3 h. Cells were then treated with 0.25 μg (6.25 μL), 0.5 μg (12.5 μL) or 1 μg (25 μL) of *Nf69*-secreted R18-labeled EVs starting at the longest timepoints and followed by shorter timepoints prior to the final 3 washes with 50 μL of PBS and fixation with 4% PFA for 15 min prior to a wash with PBS followed by staining with 10 μg/mL of Hoescht for 45 min and one final wash in PBS. Plates were then sealed with Axygen sealing film (Corning, Somerville, MA, United States; cat#:PCR-SP) prior to imaging at 20X with an ImageXpress Micro Confocal system using a DAPI filter to image Hoescht-stained nuclei and a TRITC filter to image R18-labeled membranes. The MetaXpress High-Content Image Acquisition and Analysis Software (v6.7.2.290) was used to create a custom module to analyze images and enumerate fluorescence intensity at the cell population level in a non-biased manner. Cells were detected with a cell scoring mask that first identified nuclei using the DAPI channel (size range of 2–30 μm; intensity 1,500–15,000 above background). The nuclei were then deemed positive or negative depending on the intensity above the local background surrounding the nucleus in the TRITC channel (size range of 5–50 μm; intensity of 200–425 above background). The mean integrated intensity of the fluorescent values in the TRITC channel for “positive nuclei” was calculated and the summation of these values are presented in Supplementary Figure S4. The exact intensity cutoffs for the above masking strategy were calibrated to each plate and cell line using unstained controls. Four technical replicate wells were used per treatment group and two independent biological replicates were performed.

#### 2.6.4. Amoebae R18-stained EV uptake visualization via ImageStream

Two days before assay, V631 amoebae were harvested, counted, and seeded into microcentrifuge tubes at concentrations of 50,000 cells per tube in a total volume of 1 mL of NCM supplemented with 10% EV-depleted FBS and penstrep. Tubes were gently agitated once a day to induce growth. *Nf69* EVs were stained as described earlier and each tube was treated with 1 μg of R18-stained EVs and incubated at 34°C for various timepoints. At the end of a timepoint, tubes were spun down at 14,000 rpm for 2 min and the supernatant was carefully aspirated and replaced with 500 μL of incomplete NCM. This wash process was repeated two more times prior to adding a stain/fixative mixture that consisted of 4% PFA, 0.5% glutaraldehyde and 2 μg/mL Hoescht and incubating at RT for 45 min. Tubes were spun down at 14,000 rpm for 2 min and washed with 500 μL of PBS prior to a final spin and resuspension in 100 μL of PBS. The imaging flow cytometry data acquisition template was set to collect 15,000 events at 60X magnification with channel 405 set to 10 mW, channel 561 set to 200 mW and the SSC channel set to 1 mW. Flow cytometry data was analyzed and compensated using the IDEAS software (Luminex, Austin, TX, United States; v6.2.187.0). Unstained and single-color controls were used to compensate data for each replicate. Gating was first performed to select for focused cells followed by Hoescht-positive cells as a secondary gate to exclude debris. Data was exported to FCS Express 7 Plus (*De Novo* Software, Pasadena, CA; v7.12.0007) to create histogram overlays and visualize shifts in fluorescence intensity for cell populations from each timepoint.

### 2.7. RealTime-Glo EV assay

The RealTime-Glo MT Cell Viability Assay (RTG; Promega, Madison, WI; cat#:G9712) previously described by Colon et al. and Rice et al., was used to assess whether any differences in viability of Vero, A549, or B103 cells occurred in response to exposure to Nf-secreted EVs (Colon et al., 2019; Rice et al., 2020). As shown in Supplementary Figure S5, we first determined the optimal seeding density of cells within 96-well plates for each mammalian cell line by performing dilution series testing (2,500 to 40,000 cells/well) at various timepoints (6 h, 12 h, 24 h and 36 h) using the CellTiter-Glo 2.0 assay as described in our previous publication (Russell and Kyle, 2022). Being that 5,000 cells fell within the linear range of the assay at all timepoints tested (except for HFFs which were excluded from this assay due to a lack of linearity), we selected this concentration and seeded cells with four technical replicates per concentration tested in a volume of 35 μL per well. Cells were allowed to attach and then serum-starved by adding non-supplemented base media 1–2 h prior to adding EVs. The protein concentration of freshly extracted brain passaged *Nf69* EVs was determined, and dilutions were made as necessary to allow a standard volume of 15 μL to be added to each well. The same process was followed for frozen stocks that were pulled from −80°C storage and thawed on ice for testing. Prior to adding EVs, the RTG substrate and enzyme were equilibrated to 37°C and mixed in respective base growth medias of each cell line. EVs were added to cells and 50 μL of the 2X RTG enzyme-substrate mixture was immediately added to obtain a 1X concentration. Plates were quickly sealed and incubated in a SpectraMax i3x plate reader (Molecular Devices, Sunnyvale, CA, United States) at 37°C and relative luminescence units (RLUs) were recorded every 2.5 min for 20 h. Negative/untreated controls were seeded with four replicates per cell line and included 15 μL PBS + cells, 15 μL media + cells, 50 μL media only, and 50 μL PBS only. EV only controls were included for each replicate and consisted of 1 μg of EVs (15 μL) in 35 μL of PBS. Initial dilution series testing of varying EV protein concentrations exposed to B103 cells was performed (Supplementary Figure S6) prior to proceeding with 1 and 0.5 μg. Data was analyzed and graphed using GraphPad Prism 9 (GraphPad Software, La Jolla, CA, United States; v9.5.0).

### 2.8. Proteomic characterization of EVs

#### 2.8.1. Preparation of samples

Three biological replicates of 2 L EV preps for *Nf69*-conditioned media were extracted under aseptic conditions (in biosafety cabinet, with 70% EtOH sterilized equipment, etc) and concentrated to <200 μL in PBS prior to measuring protein concentration as described above. Aliquots of 20 μg were frozen at −80°C and sent on dry ice for mass spectrometry analyses at UC Davis. The protein samples were subjected to proteolysis by using suspension-trap (ProtiFi) devices. S-Trap is a powerful Filter-Aided Sample Preparation (FASP) method that consists in trapping acid aggregated proteins in a quartz filter prior enzymatic proteolysis. Here, proteins were resuspended in 50 μL of our solubilisation buffer consisting of sodium dodecyl sulphate, 50 mM triethyl ammonium bicarbonate in water, pH 7.55. Disulfide bonds were reduced with dithiothreitol and alkylated (in the dark) with iodoacetamide in 50 mM TEAB buffer. Digestion constituted of a first addition of trypsin 1:100 enzyme: protein (wt/wt) for 4 h at 37°C, followed by a boost addition of trypsin using same wt/wt ratios for overnight digestion at 37°C. Peptides were eluted from S-Trap by sequential elution buffers of 100 mM TEAB, 0.5% formic acid, and 50% acetonitrile 0.1% formic acid. The eluted tryptic peptides were dried in a vacuum centrifuge and re-constituted in 0.1% trifluoroacetic acid. A small portion of the extract is used for fluorometric peptide quantitation (ThermoFisher, Waltham, MA, United States), to confirm the amount of peptide injected into the LCMS system.

#### 2.8.2. LC–MS

Liquid chromatographic peptide separation was done on an ultra-high pressure nano-flow Easy nLC (Bruker Daltonics, Billerica, MA, United States). Flow rate of buffers was 0.85 μL/min, on a PepSep 150 μm x 25 cm C18 column (Bruker, Billerica, MA, United States) with 1.5 μm particle size (100 Å pores; Bruker Daltonics, Billerica, MA, United States), heated to a constant temperature of 40°C; nanoESI via a ZDV spray emitter (Bruker Daltonics, Billerica, MA, United States). Mobile phases A and B consisted of water with 0.1% formic acid (v/v) and 80/20/0.1% ACN/water/formic acid (v/v/vol), respectively. Peptides were separated using a 35 min gradient: from 0–2 min increase buffer B to 5%, 2–5 min 5–10% B, 5–28 min 10–36% B, 28–35 min 80% B. This was followed by direct elution into the mass spectrometer. MS was performed on a hybrid trapped ion mobility spectrometry-quadrupole time of flight mass spectrometer (timsTOF Pro; Bruker Daltonics, Billerica, MA, United States) with a modified nano-electrospray ion source (CaptiveSpray; Bruker Daltonics, Billerica, MA, United States). In the experiments described here, the mass spectrometer was operated in PASEF mode. Desolvated ions entered the vacuum region through the glass capillary and deflected into the TIMS tunnel which is electrically separated into two parts (dual TIMS). Here, the first region is operated as an ion accumulation trap that primarily stores all ions entering the mass spectrometer, while the second part performs trapped ion mobility analysis. Data-independent analysis (DIA) was performed on a nanoElute UHPLC coupled to a timsTOF Pro. The acquisition scheme used for DIA consisted of four 25 m/z precursor windows per 100 ms TIMS scan. Sixteen TIMS scans, creating 64 total windows, layered the doubly and triply charged peptides on the m/z and ion mobility plane. Precursor windows began at 400 m/z and continued to 1,200 m/z. The collision energy was ramped linearly as a function of the mobility from 63 eV at 1/K0 = 1.5 versus cm − 2 to 17 eV at 1/K0 = 0.55 versus cm − 2.

#### 2.8.3. Data analysis

The data-independent LCMS data was analysed with Spectronaut (Biognosys) software v16. First, the Bruker LCMS DIA files were converted into htrms files using the htrms converter (Biognosys). MS1 and MS2 data were centroided during conversion, and the other parameters were set to default. First the htrms files were analyzed with Spectronaut (version: 14.0.200601.47784, Biognosys). Then, the htrms files were subjected to quantitative data analysis via direct DIA. Here, the Spectronaut software generates a directDIA library. To generate it, calibration was set to non-linear iRT calibration with precision iRT selected. We used the protein sequence database of unreviewed *N. fowleri*, rUP000444721 and the Uniprot Crap common contaminants were used. Decoy sequences were generated and appended to the original database. A maximum of two missing cleavages were allowed, the required minimum peptide sequence length was 7 amino acids, and the peptide mass was limited to a maximum of 4,600 Da. Carbamidomethylation of cysteine residues was set as a fixed modification, and methionine oxidation and acetylation of protein N termini as variable modifications. The initial maximum mass tolerances were 70 ppm for precursor ions and 35 ppm for fragment ions. A reversed sequence library was generated/used to control the false discovery rate (FDR) at less than 1% for peptide spectrum matches and protein group identifications. Decoy database hits, proteins identified as potential contaminants, and proteins identified exclusively by one site modification were excluded from further analysis.

#### 2.8.4. Functional analyses of identified proteins

The returned list of identified proteins consisted of 2,295 proteins, 2,270 of which were specific to *N. fowleri* (others included common human or bovine contaminants such as keratin, serum albumin, etc. that came from the media or the preparation process). These amoebae-specific proteins were first run through the Blast2GO (v6.0.3) pipeline (steps included: blast search, interpro classification, GO mapping, annotation, and goslim reduction) and this annotation data was associated with the protein list in Supplementary Table S2 (Conesa et al., 2005; Conesa and Gotz, 2008; Gotz et al., 2008). Because the hierarchical charts generated by Blast2GO were too complex/difficult to follow, we utilized the Panther Classification System (v17.0) to perform functional classifications and identify potential gene family enrichment (Thomas et al., 2022). Data lists were exported, and pie charts were generated in Microsoft Excel (v2212).

## 3. Results

### 3.1. Axenically cultured clinical isolates of *Naegleria fowleri* are distinguishable by size

**Figure 1:**
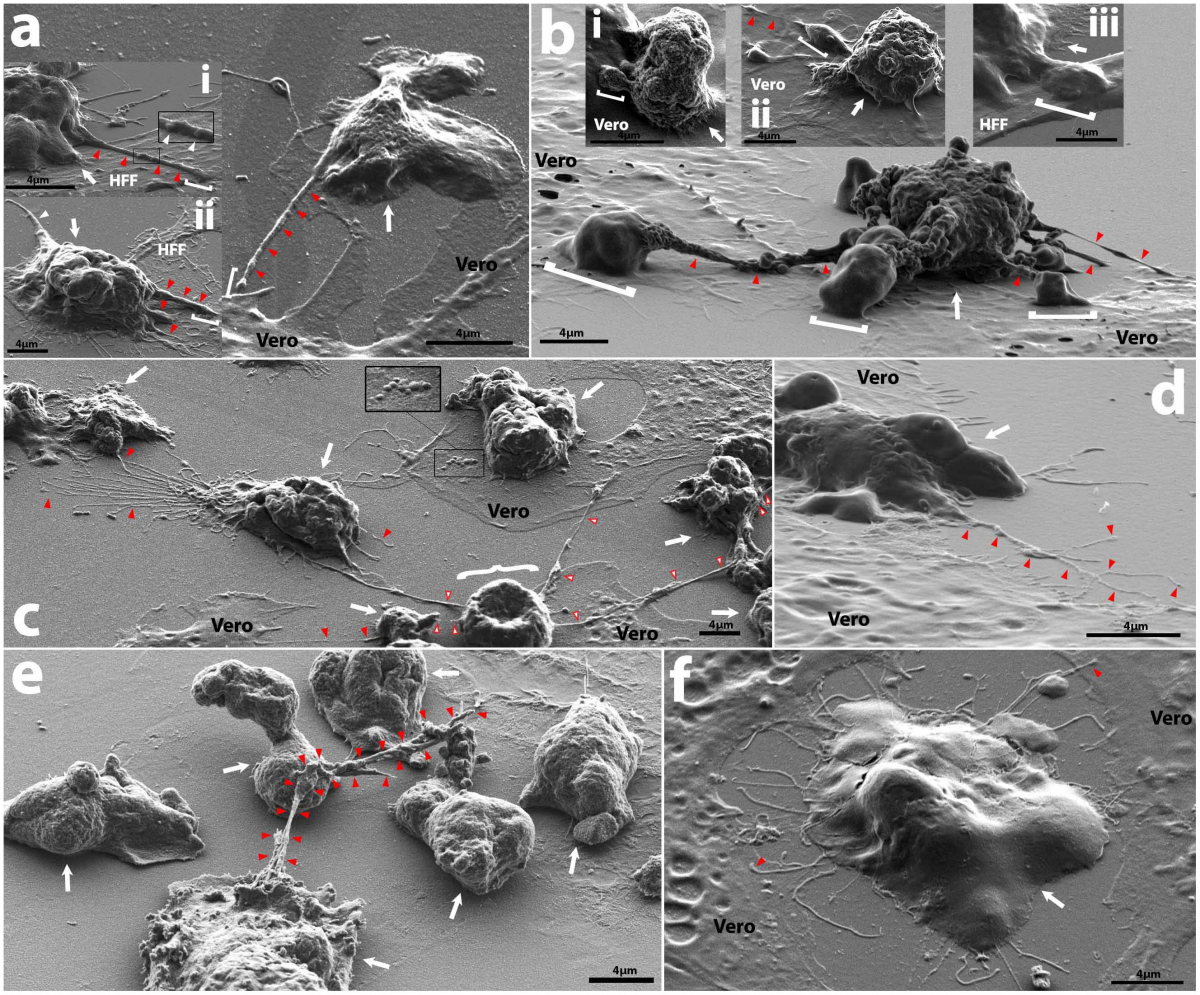
SEM micrographs of each clinical isolate in axenic culture. **(A)** *Nf69*, **(B)** *V067*, **(C)** *HB4*, **(D)** *6088*, **(E)** *V631*. **(F)** Graphical depiction of SEM measurements and EV size comparison between analysis techniques (NTA, EVs run over filter, and EVs identified on/around cells). Error bars on the charts represent the standard error of the mean. af, adhesive filament (originating from regions other than the uroid and < 300 nm); a, amoebastome; f, filopodia(>300 nm); u, uroid region; uf, uroid filaments (generally associated with uroid region and < 300 nm); p, pseudopodia; l, lobopodia; r, rough. Data shown are representative of 2 independent experiments.

We utilized SEM to visualize and compare five axenically cultured clinical isolates of *N. fowleri*. When analyzing each isolate, we made morphometric observations and took measurements of structures that are labeled throughout Figure 1 and summarized in Figure 1F. When comparing between culturing conditions (axenic or fed over mammalian cells), we found no significant differences in the average width of each individual isolate, however we did discover statistically significant differences when comparing the overall widths among isolates as determined by one-way analysis of variance (ANOVA) [*F*(4,893) = 6.983; *p* < 0.0001]. The average combined width of the five clinical isolates (composed of at least 3 measurements of length and/or width per amoeba ± s.e.m.) was 10.3 ± 0.6 μm (*n* = 898 amoebae measured), with *Nf69* being significantly larger than both *6088* (*p* = 0.0012) and *V631* (*p* = 0.0001), and *HB4* also being larger than *V631* (*p* = 0.0012; *post hoc* Tukey comparisons; Table 1; Figure 1F).

**Table 1:** Measurements of *Naegleria fowleri* amoebae and *Nf-*EVs taken with SEM and NTA (mean ± s.e.m.).

Clinical Isolate	*Nf69*	*V067*	*HB4*	*6088*	*V631*
Width (μm) *n* = number of amoebae measured	11.8 ± 0.5 (*n* = 174)	10.6 ± 0.4 (*n* = 199)	11.37 ± 0.5 (*n* = 208)	8.73 ± 0.6 (*n* = 47)	9.15 ± 0.3 (*n* = 270)
Uroid region (μm) *n* = number of measurements	5.18 ± 0.3 (*n* = 27)	3.75 ± 0.3 (*n* = 20)	3.97 ± 0.3 (*n* = 34)	n/a	3.69 ± 0.2 (*n* = 55)
Number of amoebastomes *n* = number of amoebae analyzed	0.24 ± 0.06 (*n* = 104)	0.23 ± 0.13 (*n* = 22)	0.03 ± 0.04 (*n* = 61)	0.05 ± 0.05 (*n* = 22)	0.35 ± 0.18 (*n* = 26)
Filament diameter (nm) *n* = number of measurements	137.2 ± 8.0 (*n* = 174)	172.3 ± 4.4 (*n* = 301)	212.2 ± 4.0 (*n* = 758)	156.7 ± 4.4 (*n* = 369)	173.0 ± 5.0 (*n* = 658)
EV NTA diameter (nm) *n* = number of EVs measured	150.2 ± 0.6 (*n* = 61,130)	153.8 ± 0.7 (*n* = 106,876)	157.2 ± 0.2 (*n* = 75,436)	n/a	158.9 ± 0.7 (*n* = 68,157)
EVs on filter diameter (nm) *n* = number of EVs measured	117.0 ± 1.4 (*n* = 1,369)	n/a	n/a	n/a	158.1 ± 1.8 (*n* = 817)
EVs on/near cells diameter (nm) *n* = number of EVs measured	162.7 ± 7.1 (*n* = 150)	168.4 ± 9.6 (*n* = 69)	164.2 ± 3.7 (*n* = 496)	219.2 ± 19 (*n* = 14)	130.9 ± 2.1 (*n* = 342)

We took note of one structural motif known as the uroid—an organelle previously observed on the posterior end of *N. fowleri* trophozoites with trailing filaments or processes (Carter, 1970; Visvesvara et al., 2005). It is referred to as an excretory organelle in limax amoebae (Vickerman, 1962), the potential location of the contractile vacuole in *N. fowleri* (Martinez et al., 1971), and a structure potentially involved in releasing vacuoles, waste, and excretory granules in *Entamoeba histolytica* (Hopkins and Warner, 1946). Our current work shows that the uroid of *N. fowleri* consists of clusters of hemispherical bulges of the membrane (rounded blebs or clusters of membranous invaginations) and was identified in all clinical isolates analyzed except for *6088*, although it was only visible in a subset of the amoebae. The size of the uroid regions (reported in Table 1 and Figure 1F) seemingly correlated with the width of the isolates as evidenced by the largest isolate, *Nf69*, boasting a significantly larger uroid region compared to *V067* (*p* = 0.0081), to *V631* (*p* = 0.0002), and to *HB4* (*p* = 0.0109) [one-way ANOVA and *post hoc* Tukey comparisons; *F*(3,132) = 6.540; *p* = 0.0004].

Often, we observed bundles of thin filaments with occasional bulbous tips—herein referred to as uroid filaments—that extruded from the uroid region, adhered to the substrate, and were repeatedly found broken off on either the mammalian cell or the substrate surface surrounding imaged amoebae. Other filaments with occasional bulbous tips that we suggest are adhesive filaments, often extended from the membrane around the entire cell periphery. We measured the diameters of these filaments in axenic cultures and report them in Table 1. Lastly, we have confirmed the observation made by Antonios et al. that no or very few organized “suckers” or food-cups/amoebastomes were identified on the trophozoite stages examined (Antonios, 2010). Contrary to some reports of one or more food cups per amoeba (John et al., 1984; Sohn et al., 2010), easily identifiable food cups were rare, and only identified in a small fraction of images taken of axenically cultured amoebae as well as those cultured over mammalian cells (Figures 1A,F; Table 1; Supplementary Figures S1A,D,H,J).

### 3.2. Feeding *Naegleria fowleri* produces distinct structures and induces cytopathic effects in co-cultured mammalian cells

**Figure 2:**
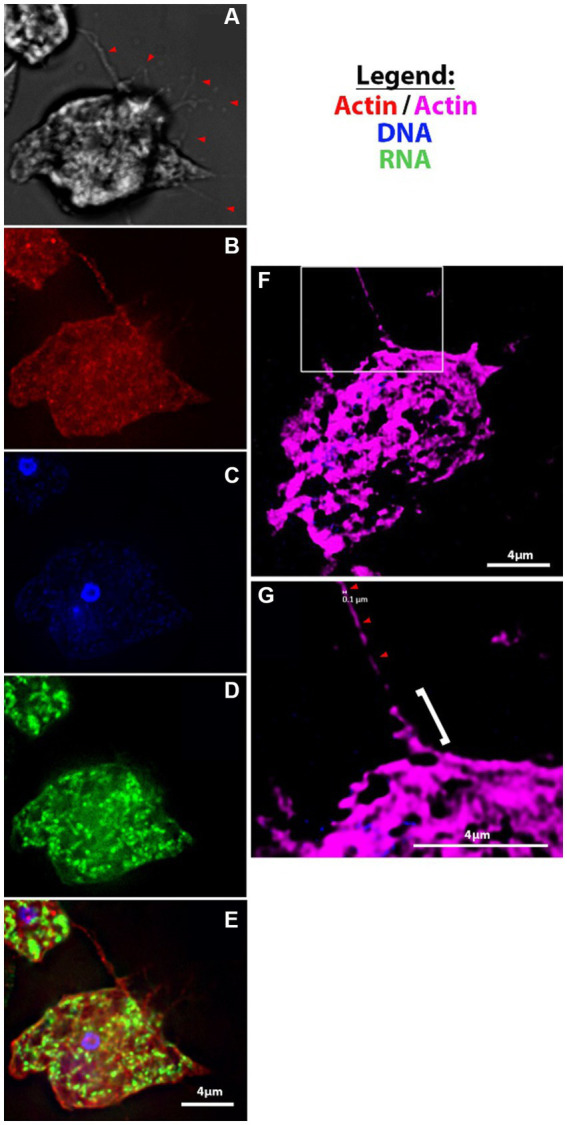
Representative SEM micrographs of intercellular interactions among amoebae and mammalian cell monolayers. **(A)** Main panel shows *V067* trophozoite (white arrow) extending a filopodium of ~300 nm in diameter (red arrowheads) a distance of ~6.3 μm away from the cell body to a nearby Vero, subpanel i shows a *V631* trophozoite (white arrow) feeding on HFF and extending filopodia of ~300 nm diameter a distance of >6.75 μm containing potential cargo of ~200 nm (white arrowheads) onto HFF, while subpanel ii shows a *V631* trophozoite (white arrow) feeding on HFF cell with 5.6 μm long uroid filament extending from rear of cell (left white arrowhead) and filopodia of varying width (right red arrowheads) reaching onto HFF cell (of which the largest varies in diameter from 600 nm to 1.1 μm and extends ~6.74 μm from cell), a white bracket shows contact point; **(B)** *V631* trophozoite (white arrow) producing pseudopodia that act as contact points with Vero cells (white brackets) and extend from the cell body via rough tubular structures (red arrowheads), also labelled with red arrowheads are uroid/adhesive filaments, subpanel i shows a *V631* trophozoite with a rough surface feeding on a Vero cell and extending a pseudopodium (white bracket), subpanel ii shows a *V631* trophozoite feeding on an HFF cell with a long filament (white arrowhead) extending from a thicker filopodium (white bracket), and subpanel iii shows a *V631* trophozoite extending a pseudopodium with raised topography onto an HFF; **(C)** *HB4* with connections to neighboring amoeba via uroid filaments (red arrows), white curly bracket shows apoptotic Vero cell with adhesive filopodia (white arrowheads w/red outline) that amoebae on right side of photo are attached to and cluster along when feeding; inset shows apoptotic blebs forming on Vero; **(D)** *Nf69* trophozoite (white arrow) on Vero cell and extending a thin, branching filament (red arrows); **(E)** axenically cultured *V631* trophozoites connected via a filopodial structure ranging from 172.6 nm to 1.27 μm a total distance of ~15.4 μm from the originating cell; **(F)** *Nf69* trophozoite (white arrow) producing filaments around the cell perimeter that make contact with neighboring Vero cells (red arrowheads).

Evidence shown by John and John (1994) indicates that allowing *N. fowleri* clinical or environmental isolates to feed over mammalian cells for multiple passages confers higher levels of pathogenicity, thus we reasoned that this mechanism could be leveraged in a controlled visual assay for understanding the structural differences arising from actively feeding versus growing axenically in culture. We exposed the amoebae to three different food sources: human foreskin fibroblasts (HFF), B103 rat neuroblastoma cells (B103), and Vero green monkey kidney cells. All isolates, except for *6088*, readily consumed HFF and Vero monolayers, while feeding on B103s was rare and commonly resulted in amoeboid encystation. Initial criteria for the selection of mammalian cells to monitor sub-micron level interactions among amoebae and their food allowed for the exclusion of B103s and HFFs as these both produce long/fibrous cellular connections and raise from the substrate at times when growing in a semi-confluent to confluent monolayer (Supplementary Figures S2A,C). Vero cells were selected as these produced uniformly flat monolayers with little to no visual fibrous intracellular connections or extracellular nanotube structures, thereby allowing for the least confounding identification and differentiation between amoebae and mammalian cells (Supplementary Figures S2E,G). Cytopathic effects were noted for all strains and cell lines when cultured with amoebae (Figure 2C; Supplementary Figure S2) and consisted of initial apoptotic bleb formation (inset in Figure 2C) followed by rounding of the mammalian cell accompanied by partial detachment from the substrate and the formation of long thin dendritic fibrils extending from main cell body. When these thin fibrils are formed, the amoebae attach to them and cluster along them (regardless of whether they are still associated with the substrate), and a common phenomenon seen when culturing over mammalian cells are networks of floating mammalian cell connections with dense clusters of amoebae dispersed along their lengths (example in right side of Figure 2C).

Prior work on *N. fowleri* by Antonios et al., described a thin surface extension when comparing the morphology of axenically cultured amoebae versus those freshly extracted from a brain and speculated that they played a role in adherence to brain tissues (Antonios, 2010). In our study, we also noted the formation of elongated thin filopodia extending from amoebae to mammalian cells (Figure 2Ai,ii). Due to their thick nature and extension over mammalian cells, we speculate that these could provide a basis for material exchange (inset in Figure 2Ai). Additionally, in an axenically cultured *V631* prep, we noted a thick filopodial extension connecting three different trophozoites (Figure 2E) which could contribute to biofilm formation. We also observed pseudopodia with raised topographies extended onto mammalian cells, sometimes with thinner tubular structures connecting them to the main cell body (Figure 2B), but mostly with the canonical fan-shaped pseudopodia (Figure 2Bi,ii,iii; Supplementary Figures S1C,F,L). We speculate that, due to the raised nature of the pseudopodia, there are likely amoebastomes or other structures facilitating material exchange being formed at the interface between the pseudopodia and the mammalian cell, but further characterization was not possible due to the limitations of SEM.

**Figure 3:**
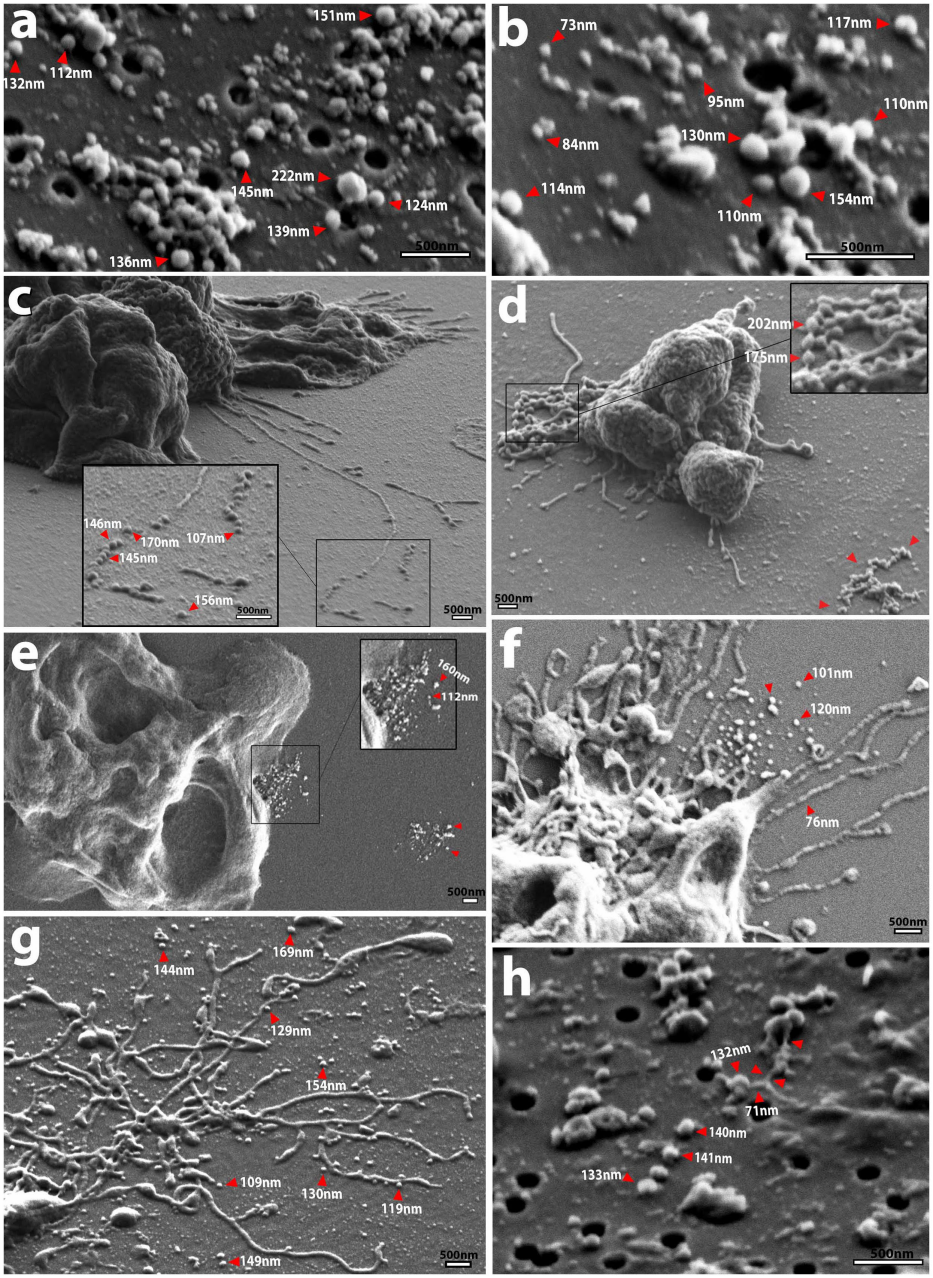
Fluorescent microscopy of filopodia and filaments of amoebae. **(A-E)** *V631* trophozoites at 100X using Deltavision II [**(A)** DIC, **(B)** Actin, **(C)** Hoescht, **(D)** 132A RNA stain, **(E)** merge]; **(F–G)** super-resolution images of actin-stained *HB4* filament of ~100 nm diameter (red arrowheads) that looks to be protruding from a thicker filopodial extension from cell body (white bracket).

Another phenomenon that we noted was the formation of thin branching filaments—sometimes emanating from the uroid region (Figures 2C,D), sometimes surrounding the entire cell periphery (Figure 2F; Supplementary Figure S1C)—that seem to be in contact with other amoebae and/or mammalian cells. We hypothesize that this could be a method of probing of the environment employed by the amoebae. We show that these thin filopodial extensions are formed via actin polymerization and can be visualized with high resolution fluorescence microscopy (Figures 3A–G; Supplementary Video S1). Moreover, we also observed the formation of thin filamentous connections from cysts to neighboring trophozoites (Supplementary Figures S1F,I). We speculate that this is either a tethering technique or a technique for intracellular signaling between cysts and/or trophozoites and the environment. The cysts exhibited the canonical rounded/oval form—sometimes with an exit pore (Supplementary Figure S1F) – and a finely reticulated membrane texture previously described by Lastovica (1974).

### 3.3. *Nf-*EVs are spherical and secreted in clusters from multiple sources

**Figure 4:**
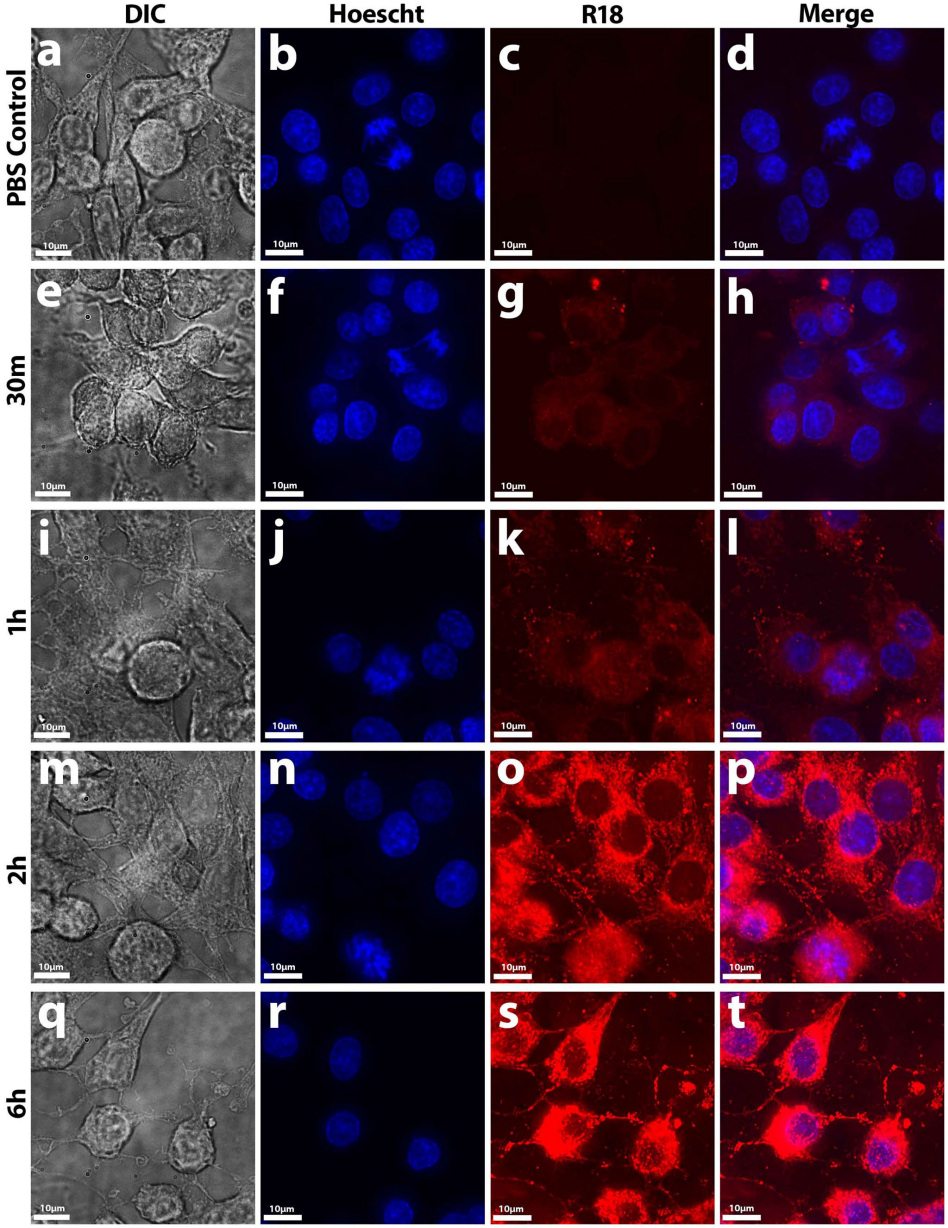
SEM micrographs showing EV shape and size as well as routes of secretion. **(A,B)** Spherical *Nf69* EVs and clusters of EVs with an avg. diameter of ~117 nm (smaller than the mean of ~140.6 nm obtained with NTA shown in Supplementary Figure S3A). **(C)** *HB4* releasing filaments that adhere to the substrate and are separating into potential EVs in a sequential beading manner. **(D)** Putative vesicles being formed in a “beads on a string” manner with successive fusion of thin filaments (red arrowheads) that ranged from ~175–200 nm. The cluster of secreted material on the right side of the panel (red arrowheads). **(E)** *Nf69* releasing clusters of EVs of various size from the periphery of the cell, potentially from a multivesicular body (MVB; red arrowheads) with another cluster on the right side of the panel (red arrowheads). **(F)** *Nf69* releasing a suspension of EVs from the uroid area of the cell. **(G)** *V631* uroid filaments surrounded by dissociated and dissociating EVs. **(H)** Example of filamentous connection of ~71 nm in diameter between EVs collected on filter from *V631* EV prep, supporting the hypothesis that extracted EVs come from both the amoeba cell membrane and adhesive/uroid filaments.

To ascertain the structure and mechanism of secretion of *Nf*-EVs, we designed an ultracentrifugation and purification technique following the Minimal Information for the Study of Extracellular Vesicles (MISEV) guidelines (Théry et al., 2018), and adapted from Bayer-Santos et al. (2013) and Szempruch et al. (2016) to extract vesicles from amoeba-conditioned media of four of the five isolates analyzed in this study. We extracted EVs from all isolates except *6088* as it was particularly sensitive to the transition from normal culture media to the EV-depleted fetal bovine serum (FBS) supplemented media. Nanoparticle tracking analysis revealed an average diameter of 152.6 nm for *Nf-*EVs across all isolates (Table 1; Figure 1F; Supplementary Figures S3A–D). Upon examination of freshly extracted *Nf69* and *V631* EV suspensions passed over a filter, we observed clusters of *Nf-*EVs with spherical morphologies (Figures 4A,B). We took measurements of individual *Nf-*EVs found on the filter, including those with easily interpretable edges and excluding those associated with clusters, and present them in Table 1. Following this, we examined the SEM images of amoebae and took measurements of potential EVs being secreted from the cell membrane or from filaments around the cell that are summarized in Table 1 and Figure 1F. Our results coincide with the range of 131–172 nm reported by Lertjuthaporn et al. (2022) and 43.88–216 nm reported by Retana Moreira et al. (2022). When comparing the sizes of measured *Nf-*EVs across the 3 measurement techniques and also among the 5 isolates, we found them to be significantly different via two-way ANOVA (across measurement techniques: [*F*(2,314,849) = 15.91; *p* < 0.0001]; among isolates: [*F*(4,314,849) = 27.42; p < 0.0001]; Figure 1F; Supplementary Table S1). In short, among the NTA preparations, *V631*, *HB4* and *V067* EVs were significantly larger than *Nf69* EVs; *V631* EVs were significantly larger than *V067* EVs; and *HB4* EVs were significantly larger than *V067* EVs. When comparing NTA to filter measurements, *Nf69* and *V631* NTA EVs were significantly larger than their counterparts measured on the filter. This could be due to clusters of EVs being detected as a single EV in NTA and our exclusion of these clusters from our manual measurements of SEM images.

To determine the potential source of the secreted EVs, we scanned our SEM images for secretions from amoebae that resembled the EVs visualized on filters and identified three probable sources (Figures 4C–G). Firstly, we observed that the uroid/adhesive filaments commonly separate into vesicles via beading or pearling (Rilla, 2021) and break off onto the substrate leaving behind clusters of material (Figures 4C,D,F,G). This source of EVs from filopodial extensions has been described for numerous types of mammalian cells (Lai et al., 2015; Mathieu et al., 2019; Rilla, 2021). To support this phenomenon in *N. fowleri*, we mined the images of *Nf-*EVs passed over filters and found an example of a filament associated with clusters of EVs in a similar manner to those that we visualized in amoebae cultures (Figure 4H). Secondly, we noted the release of clusters of EVs from the periphery of the cell membrane (Figure 4E) which could indicate emission of vesicles via multivesicular bodies (MVBs). Lastly, we observed clusters of materials being released from regions of the plasma membrane (Supplementary Figure S1D). Upon taking measurements of the adhesive/uroid filaments, the putative vesicles formed via beading of filaments, and the clusters of putative vesicles secreted from the plasma membrane, we found that the size range overlaps with measurements that we obtained via NTA and manually on filters.

### 3.4. R18-stained *Nf-*EVs are taken up by mammalian cells and other amoebae

**Figure 5:**
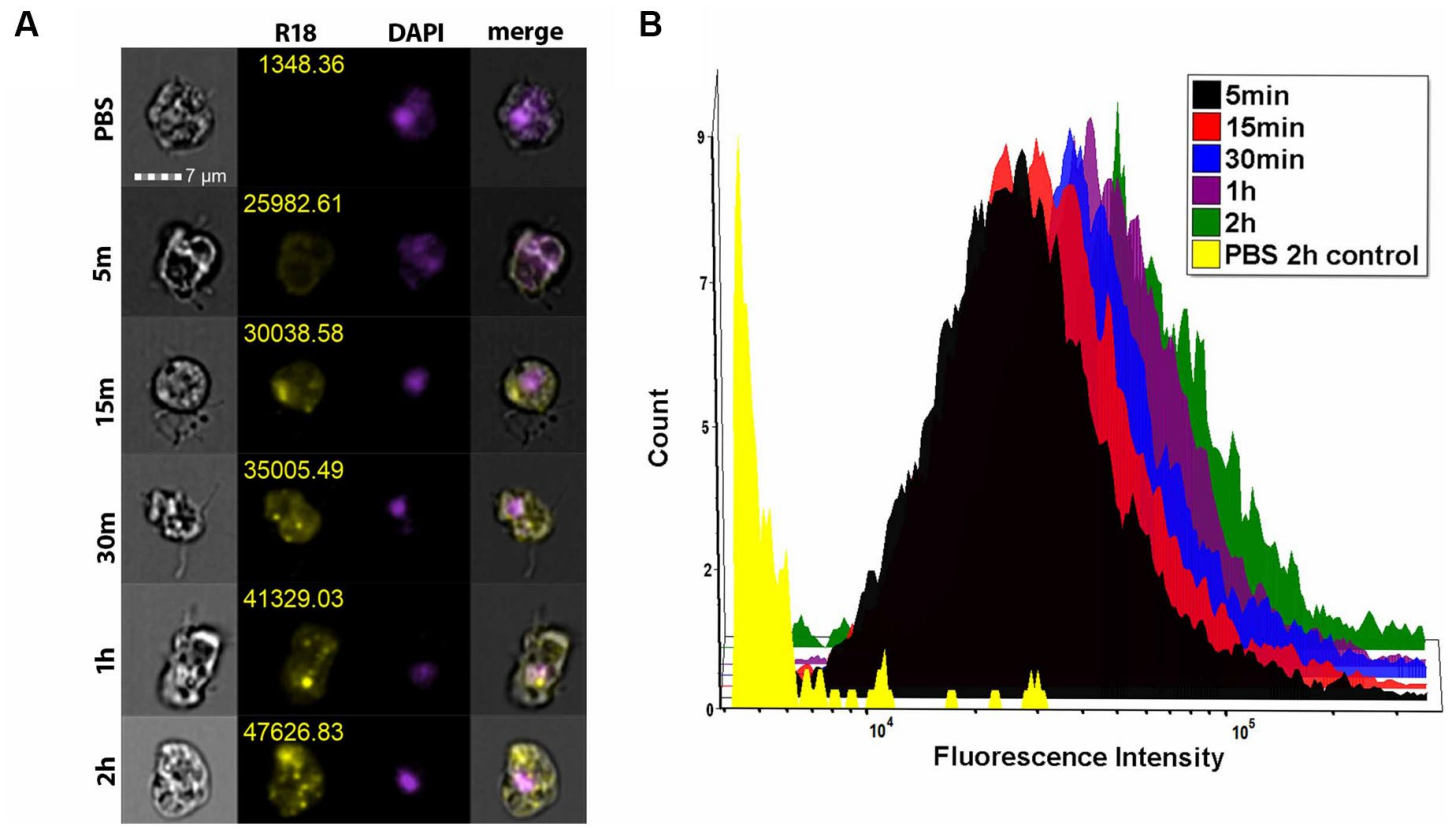
Uptake of R18-stained *Nf*-secreted EVs by B103 Rat Neuroblastoma cells. At early timepoints up to 30 min **(E–H)**, initial diffuse lipid equilibration with outer cell membranes (mainly on the cell body) occurred in treated cells compared to control samples **(A–D)**. At 1 h onward **(I–L)**, punctate staining patterns in not only the cell body but also on the axons and dendrites occurred until maximal fluorescence potential was reached at 6 h **(M–T)**. EV uptake was measured by fluorescence dequenching of R18-labeled *Nf69* EVs with mammalian cell membranes by imaging with deconvolution microscopy.

To determine whether *Nf*-EVs are taken up by mammalian cells, we labeled *Nf69-*EVs with the lipophilic fluorophore octa-decyl rhodamine B (R18) and performed fluorescence dequenching assays to monitor time-dependent increases in fluorescence that would be indicative of EV uptake by recipient cells. We first exposed B103 cells to *Nf69*-secreted R18-stained EVs for various timepoints and imaged the resulting cells (Figure 5). At the 5-min timepoint we noted initial dim lipid equilibration with B103 cell body membranes indicated by dim/diffuse staining through the 30-min timepoint (Figures 5E–H) compared to the R18-stained PBS control cells (Figures 5A–D). As more EVs were taken up by host cells over time, more of the R18 dye was diluted resulting in brighter fluorescent signals on the host cell membranes (Figures 5I–P). Initial punctate staining patterns occurred over the cell body, and this escalated to bright coverage of the full membrane (including the axons and dendrites) until fluorescence reached a plateau at ~6 h (Figures 5Q–T). We next quantified levels of *Nf69-*EV uptake for different cell lines at the population level (as a function of integrated fluorescence intensity) by using different concentrations of *Nf69-*EVs with B103, HFF, Vero, and A549 cells. We developed a 96-well plate assay using high-content imaging to detect host cell nuclei which were defined as Hoescht+ and then host cytosol defined as R18−/+. When quantifying the mean fluorescent intensity of double positive cells (host nuclei and cytosol), we observed a time and concentration-dependent increase in mean integrated fluorescence intensity per treatment group that plateaued at approximately 6–12 h (Supplementary Figure S4).

**Figure 6:**
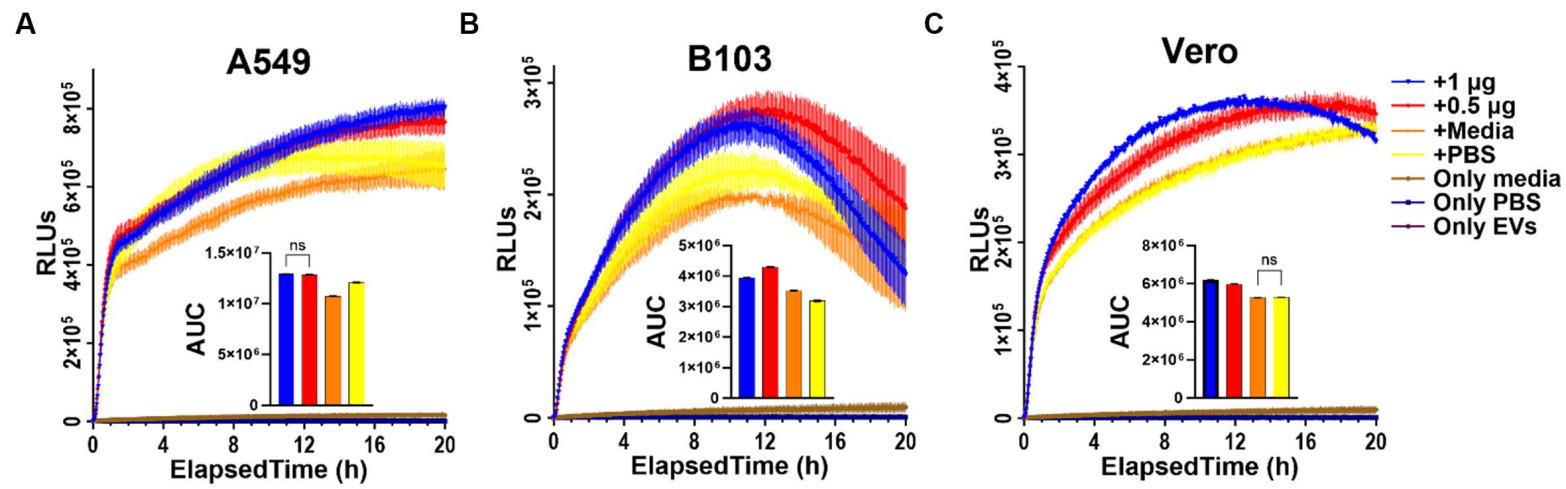
Imaging flow cytometry shows EV uptake by amoebae. **(A)** ImageStream flow cytometry data showing representative panels of mean fluorescence intensity for each timepoint. **(B)** Histogram depicting the fluorescence intensity of each of the treatment groups shows a time-dependent increase in fluorescence intensity across each treated population compared to the R18-stained PBS control.

To determine whether EVs are taken up by other amoebae, we grew the amoebae in suspension and treated *V631* with 1 μg of *Nf69*-secreted R18-stained EVs for various timepoints and performed imaging flow cytometry. We used Hoescht+ gating to select for viable amoebae followed by R18+ gating to quantify fluorescence levels of each treatment population of amoebae. Similar to mammalian cells, we saw initial rapid lipid equilibration with amoeba cell membranes (Figure 6A, 5 min) and this was followed by increasing numbers of punctate staining patterns (Figure 6A, 15 min – 2 h) indicative of vacuolized EVs taken up by the amoebae via phagocytosis with increasing levels of uptake over time (Figure 6B).

### 3.5. Exposure to *Nf-*EVs induces an increase in metabolic activity in mammalian cells

**Figure 7:**
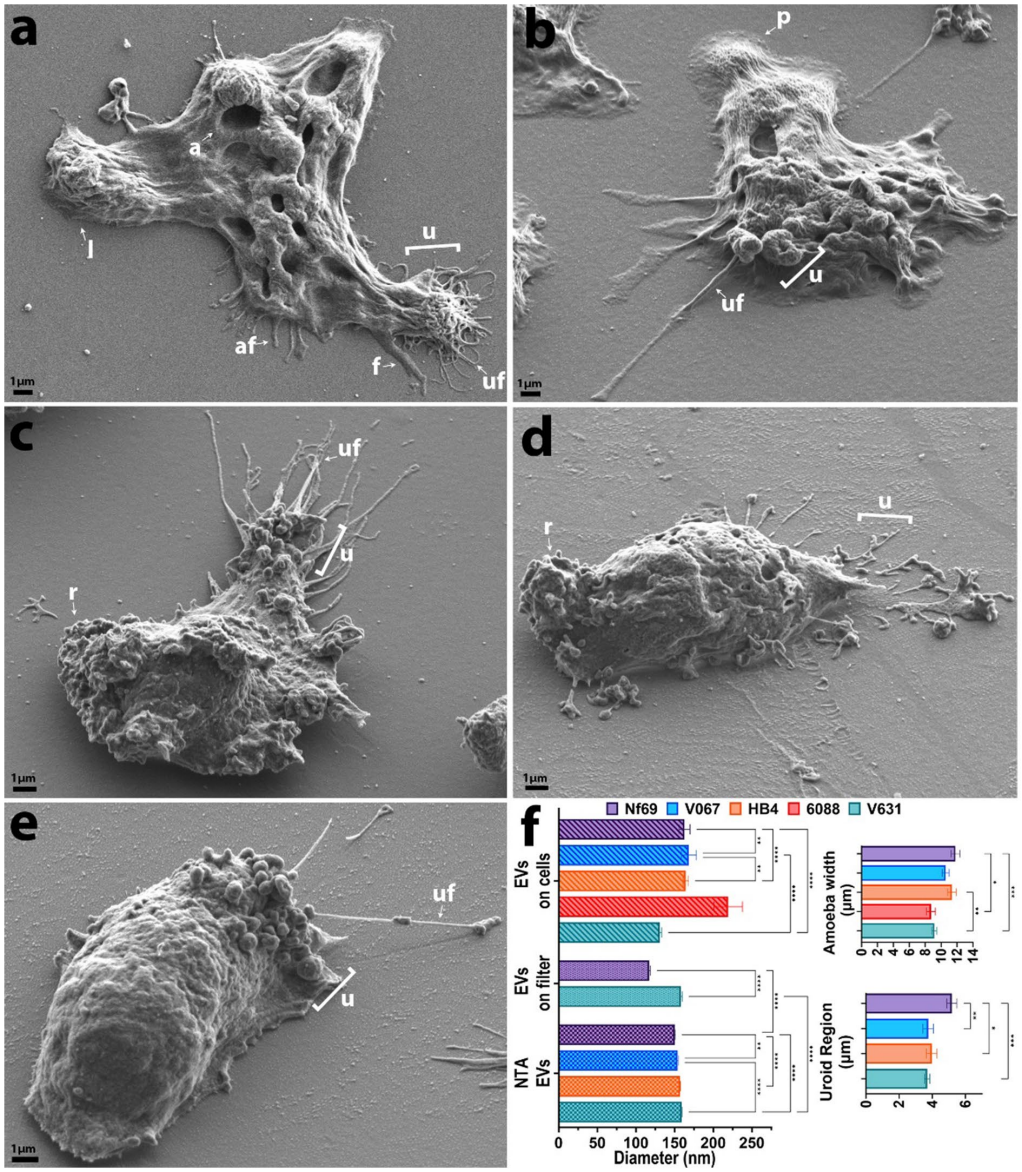
RealTime Glo results indicating increase in reducing potential in EV-treated cells. **(A)** A549 human lung carcinoma cells; **(B)** B103 Rat Neuroblastoma cells; **(C)** Vero green monkey kidney cells treated with freshly extracted *Nf69*-secreted EVs. AUC, area under the curve.

To determine if there are any measurable effects on cellular metabolism in real-time after treating cells with *Nf69*-EVs, we first optimized the seeding densities of tested cells using the CellTiter-Glo 2.0 kit in 96-well plates to confirm that the luminescence readings fell within the linear range of the RealTime-Glo MT cell viability assay at various timepoints throughout the planned incubation period (Supplementary Figure S5). The RealTime-Glo MT kit allows real-time monitoring of cell viability depending on the ability of exposed cells to take up and metabolize/reduce the prosubstrate to a substrate that is exported from the cell to bind to the NanoLuc Enzyme and produce the bioluminescence (in RLUs) that is measured as an output for the assay. We anticipated a decrease in cellular viability in response to *Nf-*EV uptake according to prior work showing that the EVs of another free-living amoeba, *Acanthamoeba castellanii,* induce cytotoxic effects in mammalian cells (Goncalves et al., 2018). On the contrary, we observed an increase in RLUs in the treatment groups compared to control groups regardless of low versus high doses, or the cell line used (Supplementary Figure S6; Figure 7). We observed the same phenomenon when we compared the efficacy of freshly extracted and processed EVs to those that were frozen immediately after processing and stored at −80°C for 14 weeks prior to thawing for the assay (Supplementary Figure S7).

### 3.6. *Naegleria fowleri* EV proteome consists of a diverse range of proteins

**Figure 8:**
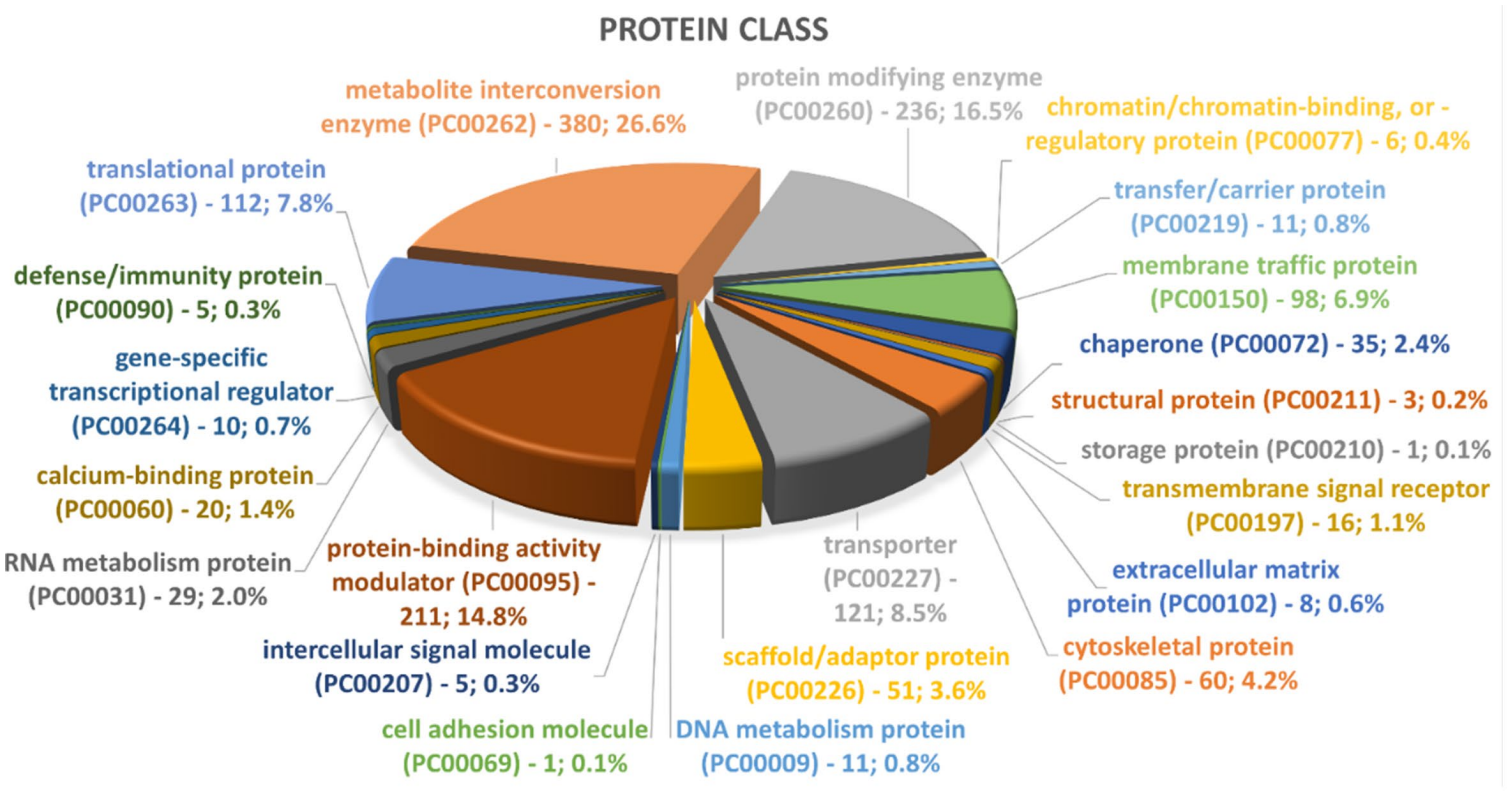
Representation of top groups of protein classes present in *Nf69* EVs. Data obtained from PANTHER analyses of the 2,270 EV proteins identified via LCMS.

To date, little is known about the protein composition of *Nf-*EVs. A preliminary visualization of protein contents of *Nf69-*EVs via SDS-PAGE gel is shown in Supplementary Figure S3E and the presence of at least 24 bands indicates that many different protein populations are present in the secreted EV proteome. To elucidate these proteins, we performed LC–MS/MS on two independent preparations of *Nf69-*EVs extracted from amoeba-conditioned media and identified 2,270 proteins present within both samples that represent the EV proteome (Supplementary Table S2). This represents a subset of 16.7% of the full 13,596 protein *N. fowleri* proteome (Zysset-Burri et al., 2014). These EV proteins were run through the Blast2GO suite, and the resulting annotations are also provided in Supplementary Table S2. We then compared our proteomic profile to the previously reported *N. fowleri* EV proteome and found that out of the 184 proteins reported by Retana Moreira et al. (2022), 18 proteins were specific to the non-pathogenic *Naegleria gruberi.* Of the remaining 166 *N. fowleri* specific proteins, 16 were not recapitulated in our study, resulting in an overlap of 150 proteins (highlighted in yellow in Supplementary Table S2) that are reiterated in our proteomic profile. We utilized the Panther classification system (Thomas et al., 2022) to identify enriched protein classes or functionalities. Out of the 2,270 proteins submitted to Panther, 1,876 were recognized for each of the 3 classification schemas. Graphical summaries of the Panther results are presented in Figure 8 and Supplementary Figures S9A,B with 1,430 protein class hits, 1,312 molecular function hits, and 2,109 biological process hits. The 5 categories in the Protein Class schema with the highest prevalence were: metabolite interconversion enzyme (26.6%; 380 proteins), protein-binding activity modulator (14.8%; 211 proteins), transporter (8.5%; 121 proteins), translational protein (7.8%; 112 proteins), and membrane traffic protein (6.9%; 98 proteins). The 5 categories in the Biological Process schema with the highest prevalence were: cellular process (38.3%; 808 proteins), metabolic process (22.0%; 465 proteins), localization (12.7%; 267 proteins), biological regulation (11.7%; 246 proteins), and response to stimulus (7.0%; 147 proteins). Lastly, the 5 categories in the Molecular Function schema with the highest prevalence were: catalytic activity (45%; 591 proteins), binding (31.5%; 413 proteins), transporter activity (5.9%; 77 proteins), molecular function regulator (5.4%; 71 proteins), and ATP-dependent activity (3.3%; 43 proteins). A direct comparison of PANTHER protein class hit categories for the entire *N. fowleri* proteome (Supplementary Figure S8) compared to hits for the *Nf*-EV proteome is provided in Supplementary Table S3.

## 4. Discussion

Ultrastructural analyses of pathogens have proven to play a significant role in the discovery of novel cell–cell interactions and provide insight into the mechanisms of cellular processes (de Souza and Attias, 2018; Caldas et al., 2020; Qian et al., 2022). Prior studies using high-resolution electron microscopy on parasitic protozoans have identified the mechanisms of extracellular vesicle secretion in *Trypanosoma cruzi* (Szempruch et al., 2016; Diaz Lozano et al., 2017; Garrison et al., 2021), monitored phenotypic effects in response to drug treatment of *Leishmania amazonensis* (de Macedo-Silva et al., 2013), and characterized the intercellular interactions between *Trichomonas vaginalis* or *Tritrichomonas foetus* and mammalian cells (Vilela and Benchimol, 2012). Thus, we utilized this technique to perform a thorough characterization of multiple clinical isolates of *N. fowleri* and EVs secreted by these amoebae. EVs are known to have a central role in intercellular communication, thus EVs and their cargo have high utility when studying infectious parasites as they have been shown to play key roles in the modulation of infection processes (Twu and Johnson, 2014; de Souza and Barrias, 2020; Drurey and Maizels, 2021). Additionally, using multiple clinical isolates of an infectious organism allows one to confirm whether specific phenotypes are conserved within the species, or potentially identify differences that could be leveraged for downstream therapeutic and diagnostic development.

In the present study, we observed significant size differences between isolates of *N. fowleri*. We also observed that actively feeding amoebae are more likely to produce filopodial or pseudopodial extensions to reach their food than their axenically cultured counterparts. Furthermore, we identified elongated filamentous structures that could aid the amoebae in communication, adherence to and steering on substrates or host cells (Xue et al., 2010), or probing the environment and searching for prey. Our observations with *N. fowleri* agree with previous studies of *Naegleria gruberi* that explore the adhesive properties and the trailing dendritic loss of cellular material as related to amoeboid locomotion (Preston and King, 1978; Preston and O'dell, 1980; King et al., 1983). We hypothesize that these are formed as the amoebae initially attach or move while adhering to different types of substrates. Furthermore, when we allowed the amoebae to adhere for longer periods of time (>3 h as compared to ~45 min-1 h), we noticed a flatter phenotype with less filament production (Supplementary Figures S1E,K). The functionality of the thicker filopodial extensions remains unknown, but we speculate that they could participate in material exchange as shown in *Trichomonas vaginalis* (Salas et al., 2023) or assist the amoebae in attaching to and feeding on bacterial biofilms. Additionally, clusters of amoebae connected by filopodia (Figure 2E) could explain our observation that amoebae float in biofilms when cultures reach high confluency, exhibit density-dependent behaviors such as cluster formation, and seemingly grow/feed faster at higher densities.

Our findings also suggest that *Nf-*EVs are released individually and in clusters and are secreted via three different mechanisms. These findings coincide with those reported for other free-living amoebae such as *A. castellanii*, which sheds vesicles from the plasma membrane (Goncalves et al., 2018), and *E. histolytica,* which sheds vesicles from MVBs and the plasma membrane (Nievas et al., 2020). Furthermore, our results with R18-stained EV uptake assays, as well as the measurement of *Nf-*EV zeta potential by Retana Moreira et al. (2022) indicate that amoebae EVs are easily taken up by numerous types of mammalian cells and other amoebae. Further experimentation utilizing plasma membrane, endosome, and lysosome markers as well as temperature controls and/or endocytosis inhibitors is warranted to provide clarification of the extent of vesicle fusion and the route of material uptake within cells. Lastly, our real-time data indicates that *Nf-*EVs induce an increase in metabolic activity in mammalian cells. We hypothesize that this could be a stress response by the mammalian cells. This metabolic response could also be explained by data from recent publications by Lertjuthaporn et al. and Moreira et al., the first showing that the EVs are immunogenic for macrophages, and the latter showing that the protein contents are immunogenic and antibodies were raised against them (Lertjuthaporn et al., 2022; Retana Moreira et al., 2022).

Very few studies have examined *Nf*-EVs and the methods used have varied significantly. Previous studies performed upon *Nf-*EVs have several shortcomings; for example, the extraction process in one study involves two separate freeze–thaw cycles (one being at −20°C for an undisclosed amount of time before EV extraction) prior to downstream analyses (Lertjuthaporn et al., 2022). This is problematic as prior EV studies have shown that conditioned media should be stored at 4°C for no longer than a week prior to processing and then samples should be stored at −80°C in aliquots that are only put through one freeze–thaw cycle to prevent degradation of EV cargoes and maintain optimal quality and enzymatic activity (Lorincz et al., 2014; Bachurski et al., 2019). Secondly, a prior study on *Nf-*EVs used a PKH26 dye to perform fusion and uptake assays (Lertjuthaporn et al., 2022), and various EV publications have shown that PKH26 dyes form contaminating aggregates (Puzar Dominkus et al., 2018; Melling et al., 2022) that must be separated from dyed EVs via sucrose gradients. This additional purification step was not performed in the prior *Nf-*EV study (Lertjuthaporn et al., 2022), thus raising the question of whether *Nf*-EVs rather than PKH26 nanoparticle contaminants are being internalized by recipient cells. In this study we utilized an extraction process that provided fresh EVs to definitively show internalization of *Nf-*EVs over a broader time range, and more accurately characterize the uptake and morphology, without destruction of membrane integrity. Lastly, another study on *Nf-*EVs (Retana Moreira et al., 2022) implemented an MISEV guided extraction process to prevent degradation, but their proteomic characterization of *Nf-*EVs consists of significantly fewer proteins (184) than we found (2,270). These differences are likely due to the short period of time (5 h) that trophozoites were incubated in media, and the lower volume of media (10 mL) used to extract EVs (Retana Moreira et al., 2022).

While SEM is powerful in deducing three-dimensional micron-level surface characteristics over a considerable area, it has limited capabilities in determining the fine microstructure of internal cellular components (Carr, 1971; de Souza and Attias, 2018). This drawback could have hindered our ability to fully explore the interaction and secretion mechanisms proposed in this study. Future studies using TEM could provide valuable mechanistic insights into the intercellular interactions and allow for definitive determination of the mechanisms of EV secretion described in this study. Being that our data has implicated the uroid structure as a key player in the secretion of materials, future functional analyses should be performed to determine whether it could assist in feeding (Chapman-Andresen, 1977) or in pathogenicity as it releases materials into the CNS that could contribute to the intense immune response associated with the disease. Despite limitations of some studies, the data on *Nf-*EVs obtained thus far warrant additional studies to establish the responses elicited by *Nf-*EVs extracted from amoebae cultured with various food sources and with differing stressors. The latter could yield significant results according to evidence that EVs secreted by *E. histolytica* contribute to parasite:parasite communication and that the amoeboid encystation process is modulated according to whether populations were exposed to EVs extracted from logarithmically growing trophozoites or encysting cells (Sharma et al., 2020). Furthermore, research exploring the effects of exposure to EVs secreted by more or less pathogenic *N. fowleri* amoebae as well as other amoebae species should be explored. Ongoing studies to characterize the RNA contents of *Nf-*EVs as well as the potential effects of EV exposure to other amoebae are being carried out in our laboratory.

## Data availability statement

The datasets presented in this study can be found in online repositories. The names of the repository/repositories and accession number(s) can be found in the article/Supplementary material.

## Author contributions

AR: Conceptualization, Investigation, Writing – original draft, Writing – review & editing, Data curation, Formal analysis, Methodology, Validation, Visualization. PB: Formal analysis, Investigation, Methodology, Validation, Visualization, Writing – review & editing, Resources, Supervision. GG: Investigation, Methodology, Resources, Writing – review & editing, Data curation. DK: Investigation, Resources, Writing – review & editing, Conceptualization, Funding acquisition, Project administration, Supervision, Writing – original draft.

## Funding

The author(s) declare financial support was received from the Georgia Research Alliance (D.E.K.) and the NIH (T32 AI060546, A.C.R; R03 AI141709, D.E.K.).

## Conflict of interest

The authors declare that the research was conducted in the absence of any commercial or financial relationships that could be construed as a potential conflict of interest.

## Publisher’s note

All claims expressed in this article are solely those of the authors and do not necessarily represent those of their affiliated organizations, or those of the publisher, the editors and the reviewers. Any product that may be evaluated in this article, or claim that may be made by its manufacturer, is not guaranteed or endorsed by the publisher.
