# Supplementary material for: Characterization of the extracellular vesicles, ultrastructural morphology, and intercellular interactions of multiple clinical isolates of the brain-eating amoeba, Naegleria fowleri
Source: Front Microbiol. 2023 Sep 27;14:1264348. doi: 10.3389/fmicb.2023.1264348 (PMC10558758; doi:10.3389/fmicb.2023.1264348)
Supplement: Supplementary file 5 [file Data_Sheet_1.pdf]

Supplemental Materials

Characterization of the extracellular vesicles, ultrastructural morphology, and intercellular interactions of multiple clinical isolates of the brain-eating amoeba, *Naegleria fowleri*

A. Cassiopeia Russell[1,2], Peter Bush[3], Gabriela Grigorean[4], Dennis E. Kyle[1,2,5]*

[1]Center for Tropical and Emerging Global Diseases, University of Georgia, Athens, Georgia, USA
[2]Department of Infectious Diseases, University of Georgia, Athens, Georgia, USA
[3]School of Dental Medicine, University at Buffalo, Buffalo, New York, USA
[4]Proteomics Core Facility, University of California, Davis, California, USA
[5]Department of Cellular Biology, University of Georgia, Athens, Georgia, USA

Data Repository: Russell, Antoinette (2023), "Supplemental Information - Characterization of the extracellular vesicles, ultrastructural morphology, and intercellular interactions of multiple clinical isolates of the brain-eating amoeba, *Naegleria fowleri*", Mendeley Data, V2, doi: 10.17632/tvvtbcvy5k.2

Supplemental Figure 1: Additional scanning electron microscopy (SEM) micrographs of amoebae

Supplemental Figure 2: SEM micrographs of mammalian cells and cytopathic effects induced by amoebae

Supplemental Figure 3: Initial Nanoparticle Tracking Analysis and SDS-PAGE analyses of *N. fowleri* extracellular vesicles (*Nf*-EVs)

Supplemental Figure 4: High-content imaging R18 *Nf*-EV uptake assay

Supplemental Figure 5: Optimal seeding densities of mammalian cells for RealTime-Glo MT cell viability assay

Supplemental Figure 6: Initial RealTime-Glo MT dilution series testing of varying protein concentrations of *Nf*-EVs

Supplemental Figure 7: RealTime-Glo MT cell viability results with frozen *Nf*-EVs

Supplemental Figure 8: PANTHER Protein Classes of proteins in entire *N. fowleri* proteome

Supplemental Figure 9: PANTHER Molecular Function and Biological Processes of proteins in *Nf*-EVs

Supplemental Table S1: Statistical Comparison Results for *Nf*-EV Measurements

Supplemental Table S2: *Nf*-EV Proteome Results

Supplemental Table S3: Comparison of protein class hits for entire *N. fowleri* proteome versus *Nf*-EV proteome

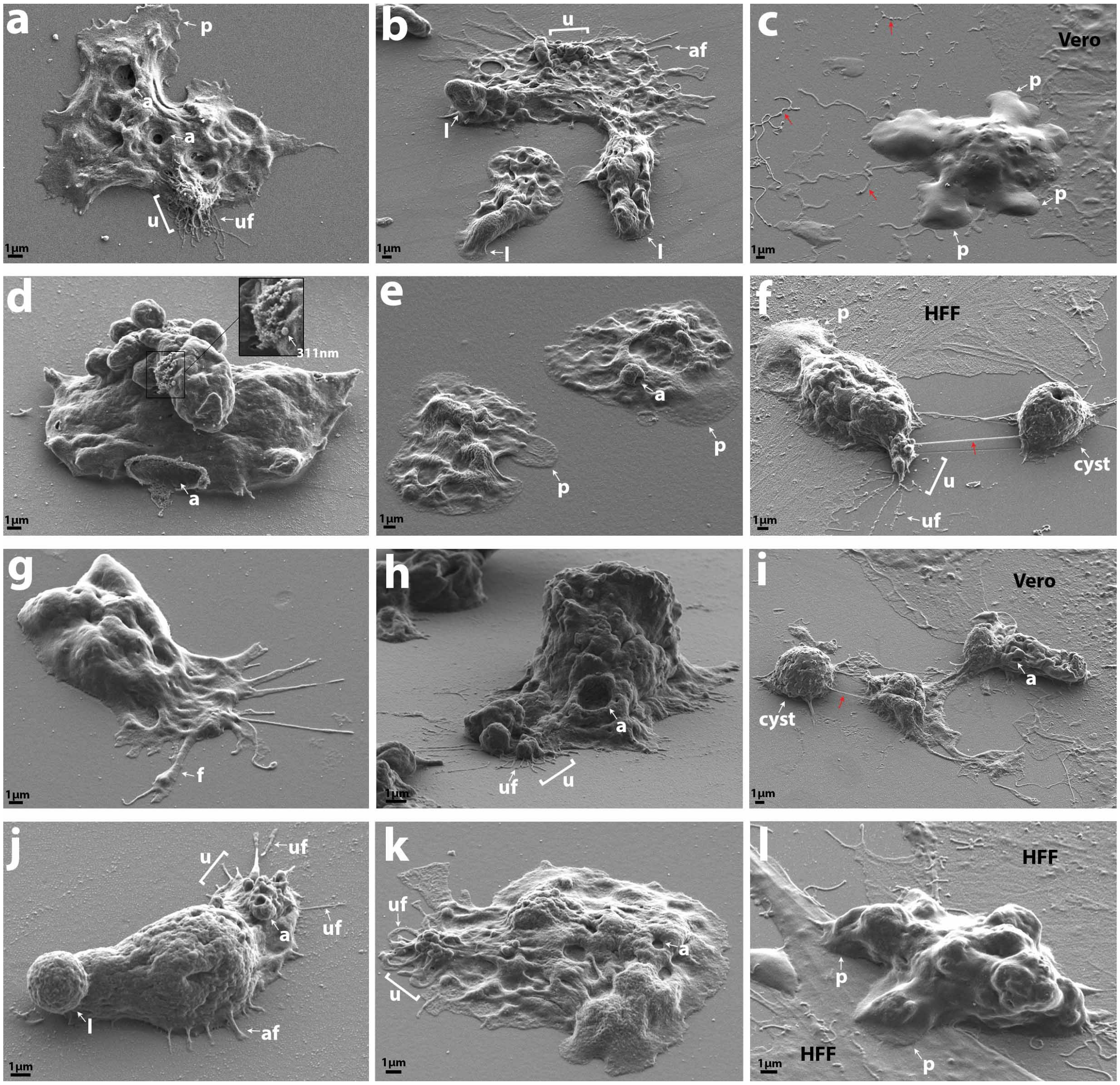

Supplemental Figure 1: Multiple micrographs of each isolate from independent experiments show repeated structural motifs that occurred regardless of the isolate being imaged and show a lack of consistency in morphology due to the amoeboid structure. Axenically cultured *Nf69* trophozoites (**a-b**) exhibiting lobopodia [l], pseudopodia [p] and bundles of filaments and membranous invaginations associated with uroid region [u, white bracket]. *Nf69* trophozoite on Vero cell (**c**) surrounded by networks of filaments around cell periphery either still attached to the trophozoite or already broken off onto the substrate (red arrows); Axenic *V067* trophozoite (**d**) with cluster of seemingly secreted matter in one region of the membrane that looks similar to the EVs imaged on the filter (in Figure 3a, b and h). (**e**) Example of *V067* in a flattened, highly adherent state. (**f**) *V067* trophozoite on HFF with a defined uroid region and trailing uroid filaments (lower red arrow) that is attached via a thin filament (upper red arrow) to an amoeboid cyst; (**g**) Example of *HB4* in an highly adherent state with a polarized region of membrane filaments; (**h**) *HB4* trophozoite in a less adherent state imaged with a 65° tilt featuring a prominent amoebastome and

numerous small filaments extruding from uroid region; (**i**) Two seemingly connected *HB4* trophozoites with a neighboring cyst/encysting amoeba attached via thin filament (red arrow); *V631* trophozoites in a less adherent state (**j**) and a more adherent state (**k**), both with recognizable uroid regions; (**l**) *V631* trophozoite feeding on HFFs via pseudopodial extensions. u=uroid, f= filopodia, a= amoebastome, p=pseudopodia.

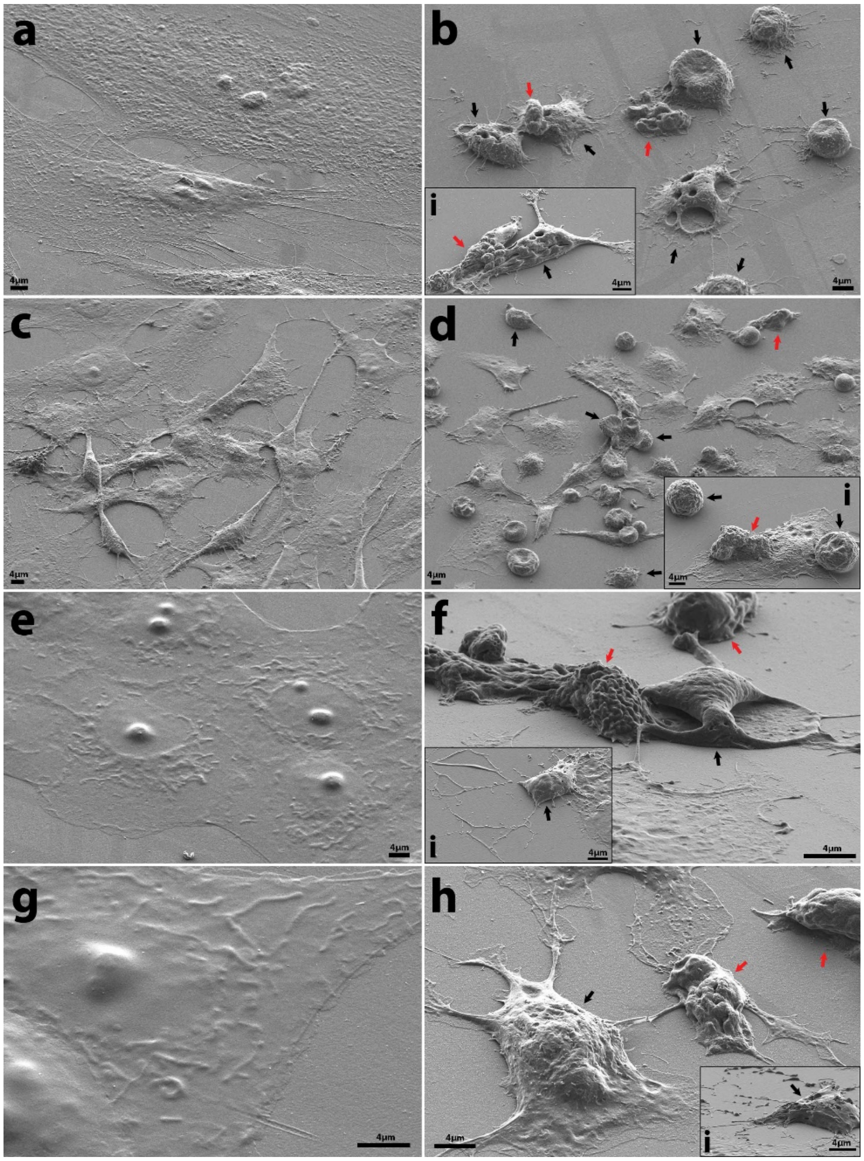

Supplemental Figure 2: Example SEM micrographs of phenotypic differences among control mammalian cell lines and examples of cytopathic effects (CPE). Mammalian cells (black arrows) are labeled as are co-cultured amoebae (red arrows). Note the long fibrous morphology and nanotube creation by HFFs (**a**) and B103s (**c**), which lead to the selection of Vero cells (**e** and **g**) for the majority of the study of intercellular interactions due to their uniformly flat nature and easily identifiable intracellular characteristics (such as the prominent nucleus and internal microtubules) that can be differentiated from amoeboid structures. (**b**) HFFs undergoing CPE while *V631* were fed over the monolayer, (**bi**) *V067* feeding on HFF and inducing CPE- note the characteristic clusters of mbs on the feeding extensions produced by the amoeba. (**d** and **di**) B103s experiencing CPE and potentially encysting *V631* amoebae (which were quite difficult to differentiate). (**f** and **h**) *HB4* amoebae feeding on Veros and inducing CPE. (**fi** and **hi**) *Nf69* induced-CPE on Vero cells.

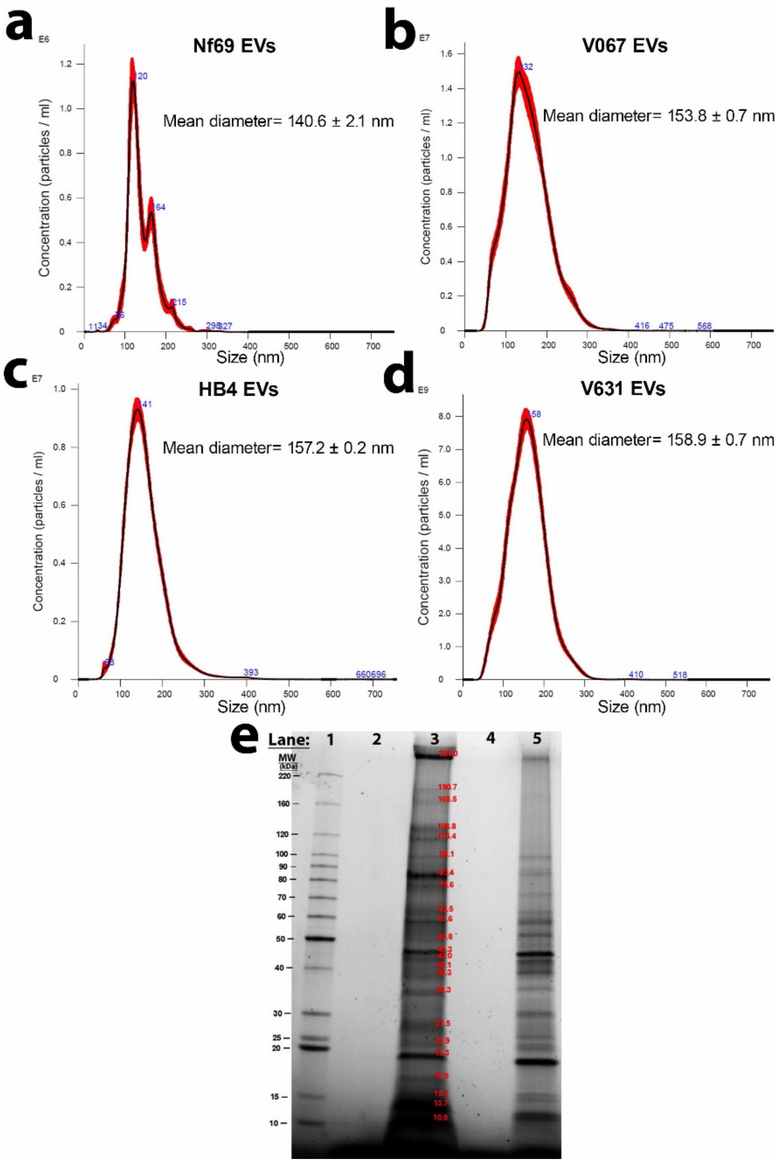

Supplemental Figure 3: *Nf*-EVs NTA and SDS-PAGE results. Nanoparticle tracking analysis results for EVs extracted from: (**a**) 1.1 L of conditioned *Nf69* media (six 225 cm^2 flasks combined) in which amoebae were grown for 118 h to a final count of $3.82x10^8$ cells resulting in a suspension of $6.15e^9$ +/- $9.16e^5$ particles/mL(1 µL diluted to 1 mL in PBS from a 250 µL suspension of EVs total vol.; detection threshold= 4), (**b**) 800 mL of conditioned *V067* media (four 225 cm^2 flasks combined) in which amoebae were grown for 312 h to a final count of $2.1x10^8$ cells resulting in a suspension of $1.76e^{11}$ +/- $6.99e^9$ particles/mL (1 µL diluted to 1 mL in PBS from a 250 µL suspension of EVs total vol.; detection threshold= 5), (**c**) 1 L of conditioned *HB4* media (five 225 cm^2 flasks combined) in which amoebae were grown for 336 h to a final count of $3.39x10^8$ cells resulting in a suspension of $8.66e^8$ +/- $8.92e^8$ particles/mL (1µL diluted to 1 mL in PBS from a 230 µL suspension of EVs total vol.; detection threshold=5), and (**d**) 1 L of conditioned *V631* media (five 225 cm^2 flasks combined) in which amoebae were grown for 192 h to a final count of $7x10^8$ cells/mL resulting in a suspension of $8.72e^{11}$ +/- $2.82e^{10}$ particles/mL (a 1:100 dilution was performed with 10 µL with a subsequent 1:10 dilution from a 250 µL suspension of EVs total vol.;

detection threshold=4). (**e**) Initial EV proteome visualization shown via SDS-PAGE with lane contents as follows: **1-**Ladder, **2-**blank, **3-***Nf69* EV sample from 2 L EV preparation sent for LC-MS/MS, **4-**blank, **5-***Nf69* cell lysate from 25,000 trophozoites exposed to repeated freeze-thaws to induce lysis.

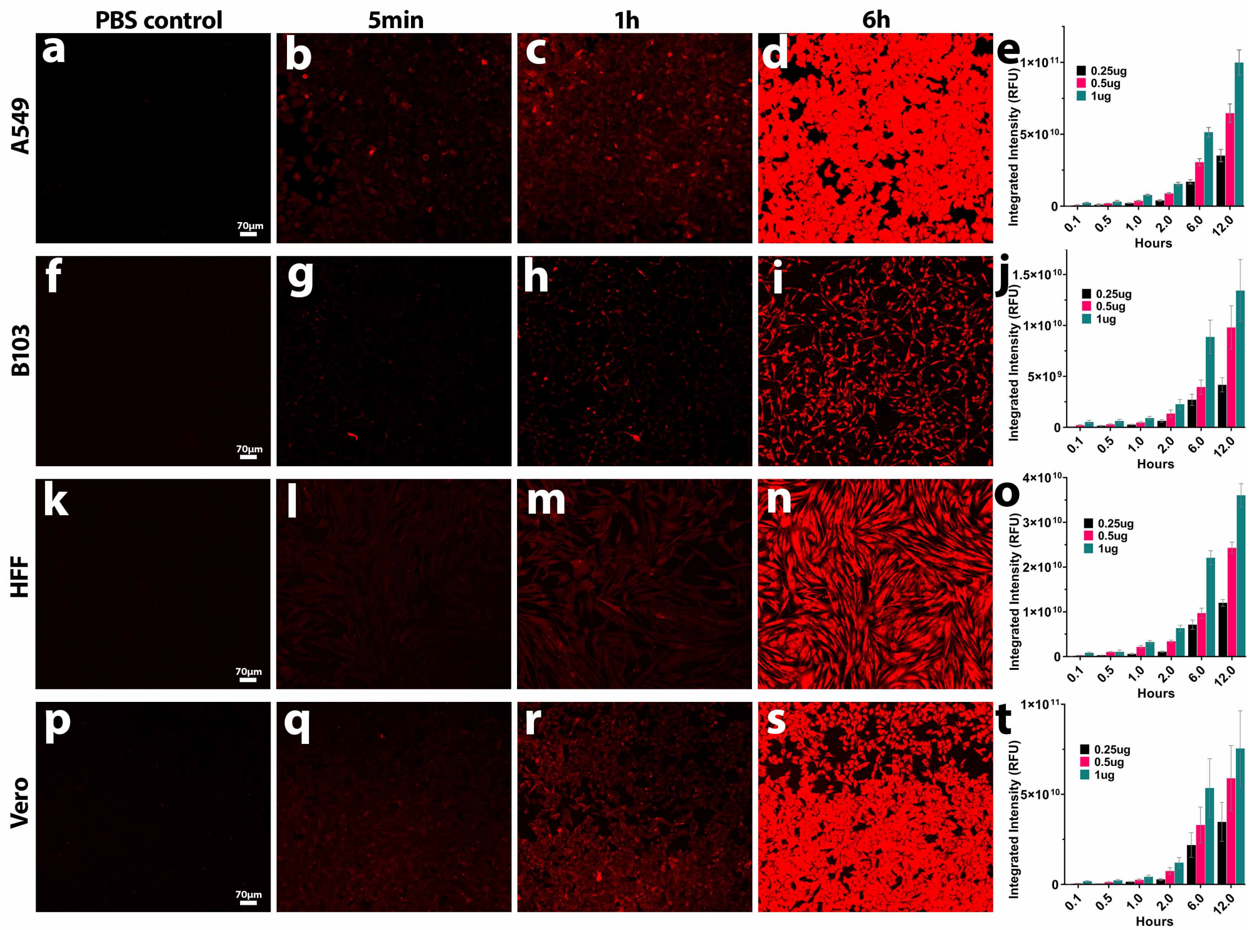

Supplemental Figure 4: High-content imaging R18 EV uptake assay. Representative selections from 1µg treatment groups per timepoint as follows- (**a-e**) A549 human lung carcinoma cells; (**f-j**) B103 Rat Neuroblastoma cells; (**k-o**) Human Foreskin Fibroblasts; (**p-t**) Vero green monkey kidney cells. EV uptake was measured by fluorescence dequenching of R18-labeled *Nf69* EVs with mammalian cell membranes. Cells were treated with three concentrations (0.25, 0.5 and 1µg of protein) of amoeba EVs and incubated for various timepoints. Plates were washed 3x, stained/fixed, and imaged using an ImageXpress Micro Confocal system. Each graph is representative of n=4 technical replicates per condition and n=2 separate biological replicates.

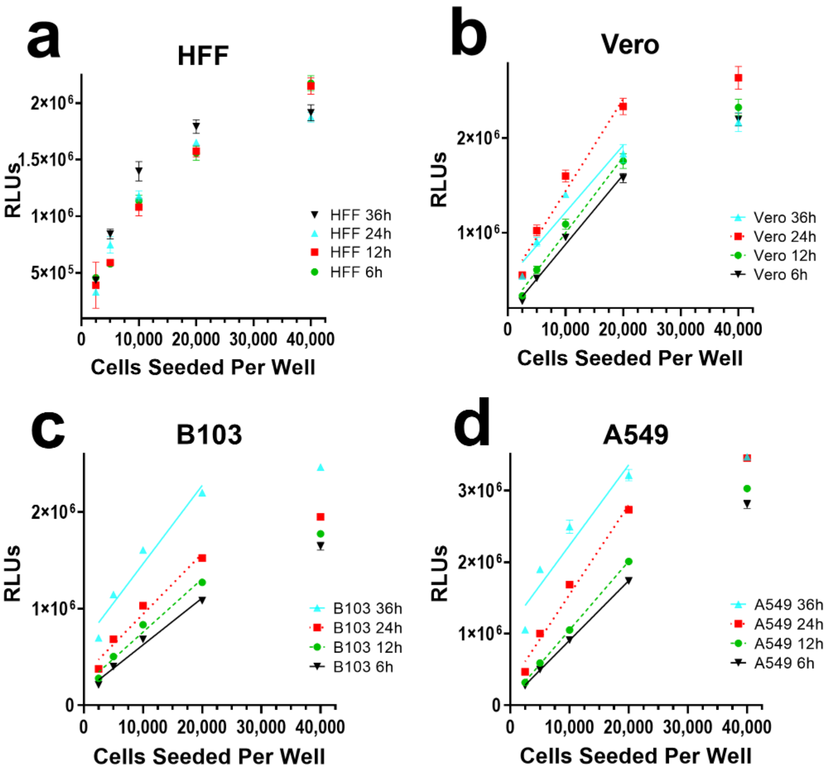

Supplemental Figure 5: Optimal seeding density using the CellTiter-Glo 2.0 assay with various seeding densities and at various timepoints for cell lines of interest. HFFs were excluded from the RealTime-Glo MT cell viability assay due to a lack of linearity (**a**), and a concentration of 5.000 cells/well was selected for each of the cell lines to remain within the linear portion of the assay (**b-d**) and also to maintain consistency.

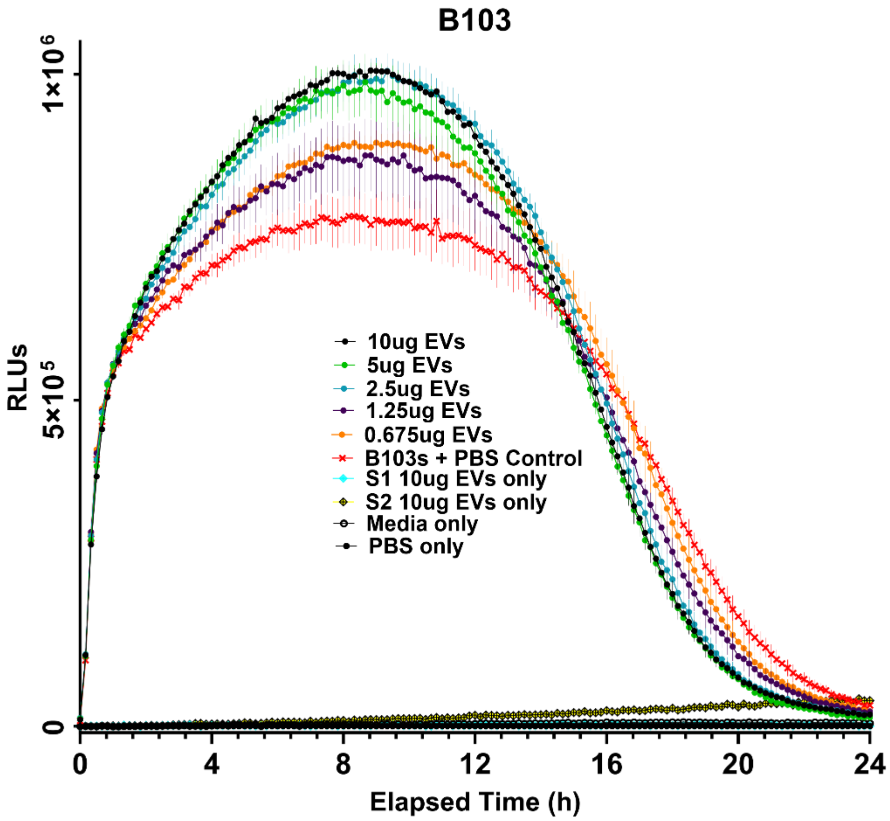

Supplemental Figure 6: Initial RealTime-Glo MT dilution series testing of varying protein concentrations of *Nf*-EVs exposed to B103 rat neuroblastoma cells. This initial data indicates an increase in RLUs across treatment concentrations compared to unexposed cells.

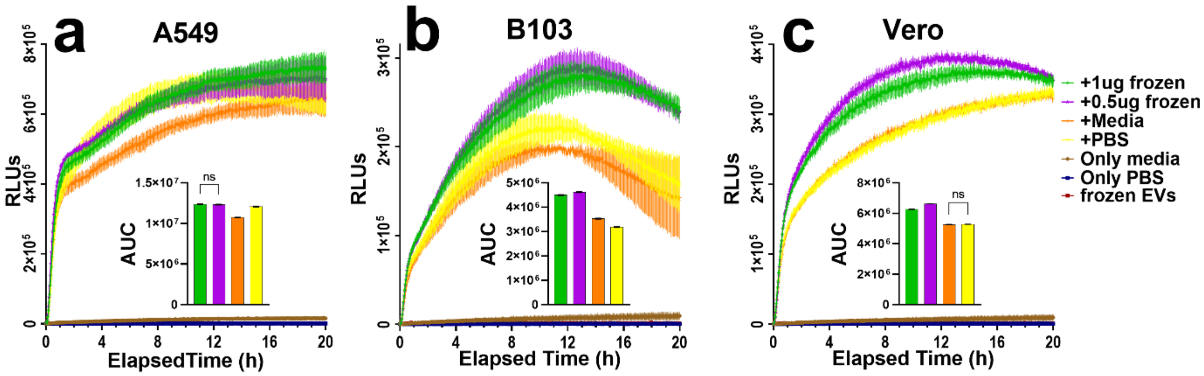

Supplemental Figure 7: RealTime Glo results with mammalian cells treated with *Nf69*-secreted EVs that were frozen and stored at -80°C showed similar results to fresh EVs. This is a direct comparison to the results with freshly extracted *Nf*-EVs presented in Figure 7 in the main text. Area under the curve charts show that there are significant differences between all curves other than those annotated "ns". Error bars on curves are representative of four technical replicate wells per treatment and cell line.

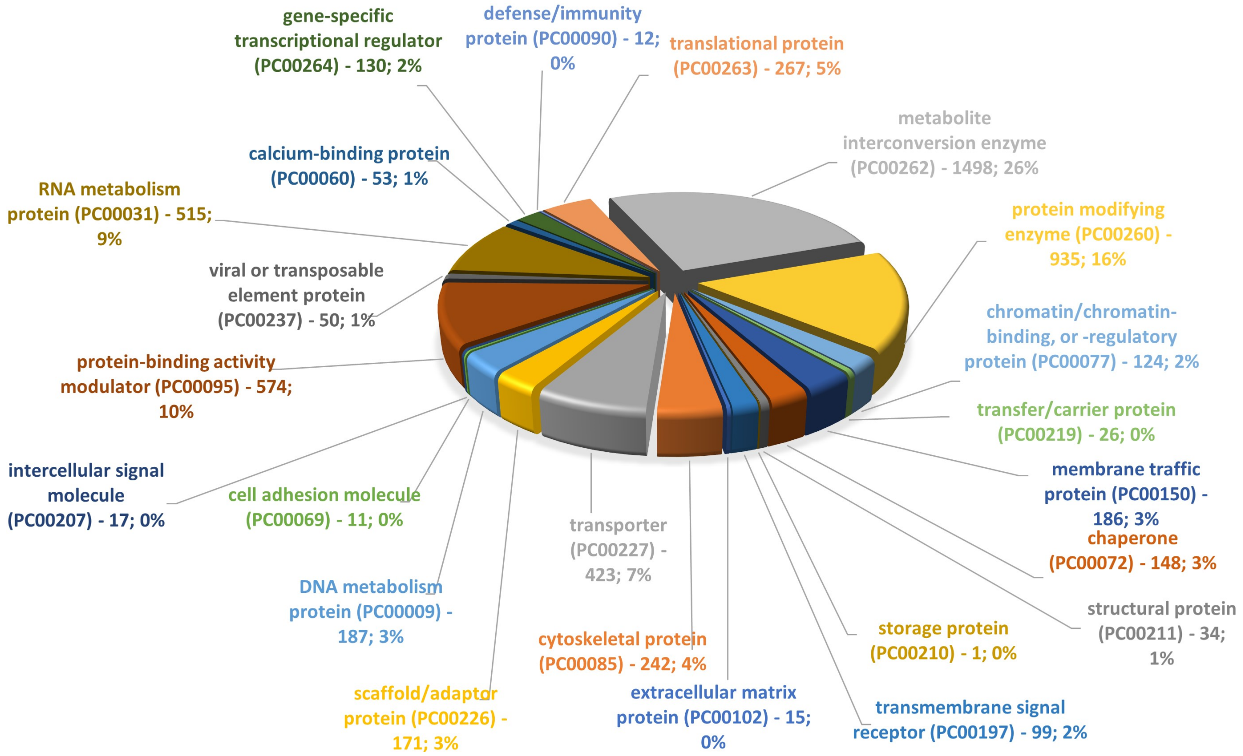

Supplemental Figure 8: PANTHER Classification System protein classes determined for entire *N. fowleri* proteome consisting of 13,742 proteins. Out of these, 8,420 were recognized within the software with 2,702 of these returning “No PANTHER Category” leading to 5,718 protein class hits obtained for the entire *N. fowleri* genome.

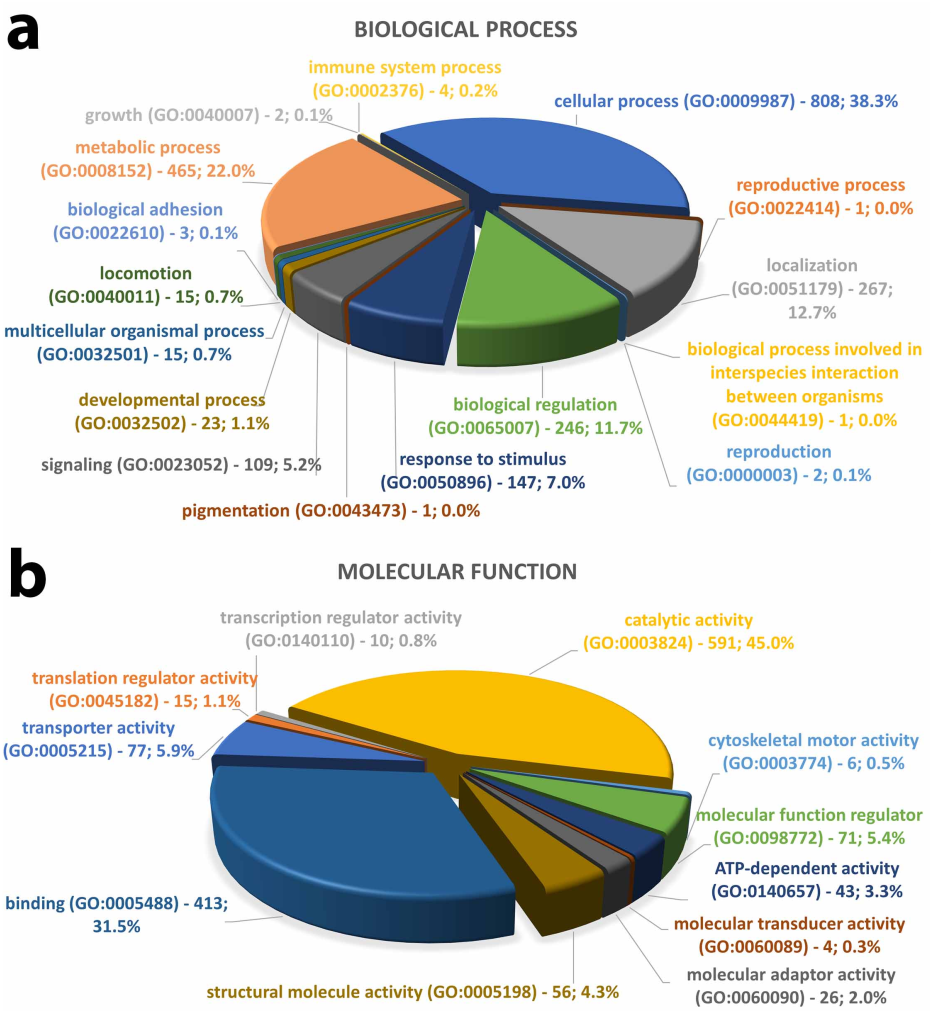

Supplemental Figure 9: PANTHER Classification System analyses of *Nf69*-secreted EV proteome. Includes biological process results (**a**), and molecular function results (**b**).

Supplemental Tables

Supplemental Table S1: Statistical comparisons for measurements of *Nf*-EVs from multiple isolates and multiple sources.

Supplemental Table S2: *Naegleria fowleri* EV Proteome obtained from *Nf69* EVs sorted from highest to lowest number of peptide occurrences. Blast2GO annotations, InterPro GO names, relative quantities obtained from each replicate sent for LC-MS/MS, the percentage coverage attained for each identified protein, and the number of peptide occurrences observed in the spectra are provided. Highlighted cells represent proteins that overlap with previously reported *Naegleria fowleri* EV proteins.

Supplemental Table S3: Direct comparison of protein numbers and percentages in protein classes determined by PANTHER of the previously published *N. fowleri* proteome versus the *Nf69* EV proteome reported in this manuscript.
